# Supplementary material for: The function of ER-phagy receptors is regulated through phosphorylation-dependent ubiquitination pathways
Source: Nat Commun. 2023 Dec 15;14:8364. doi: 10.1038/s41467-023-44101-5 (PMC10724265; doi:10.1038/s41467-023-44101-5)
Supplement: Supplementary file 1 — Supplementary Information [file 41467_2023_44101_MOESM1_ESM.pdf]

Supplementary figure 1

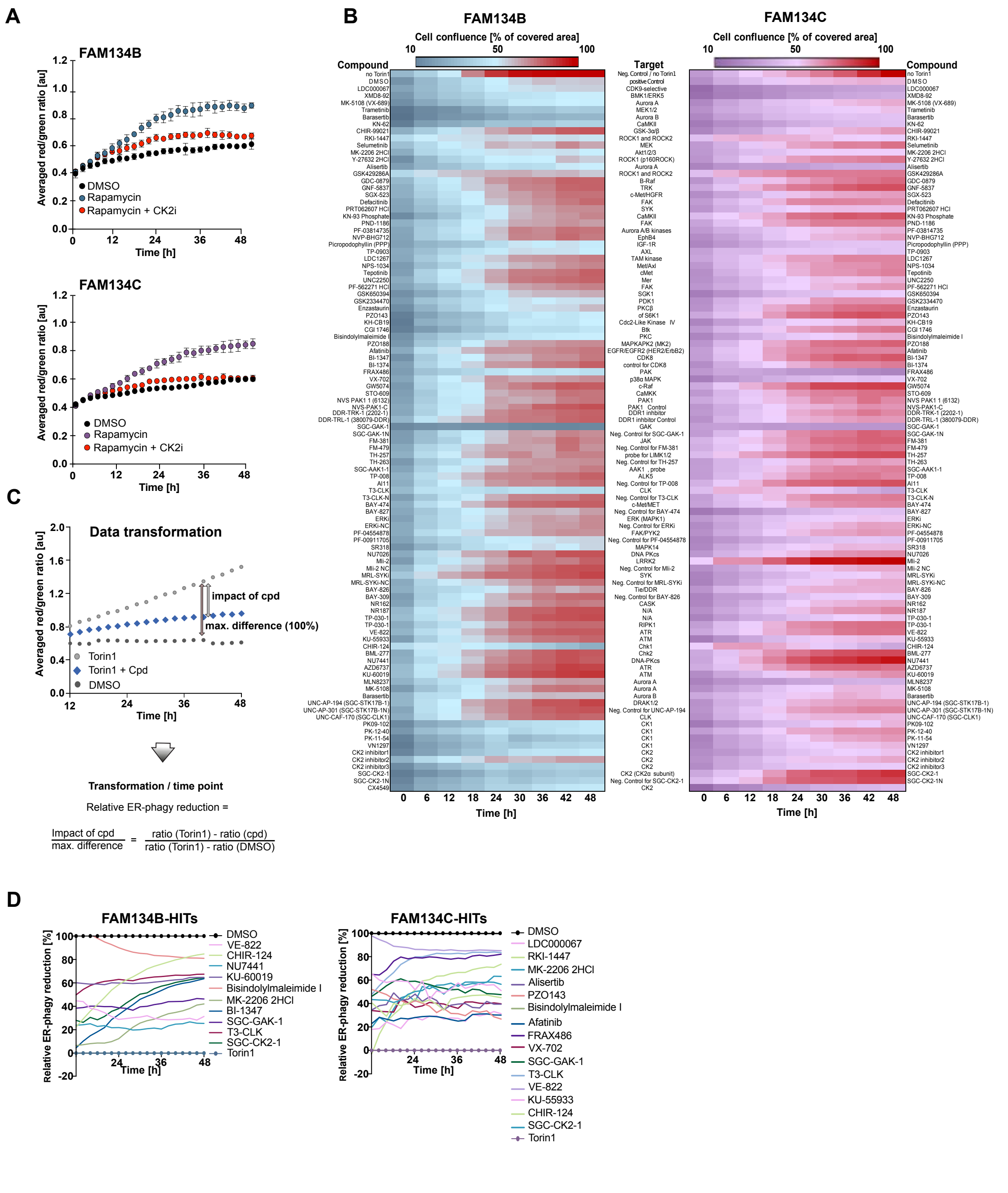

Supplementary figure 1. Identification of kinases involved in FAM134B/C and mTOR-regulated ER-phagy

(A) ER-phay flux in ssRFP-GFP-KDEL U2OS cells overexpressing FAM134B or FAM134C in basal (DMSO), autophagy-induced (40nM Rapamycin) condition in monotreatment or in combination with CK2i. Total integrated fluorescent intensities (RFP/red and GFP/green) were monitored in the IncuCyte® S3 over a time course of 48h. Data represent averaged data

obtained from n=3 individual wells via the IncuCyte® S3, each view containing > 100 cells. Data are mean ±SD, [au]: arbitrary unit. (**B**) Heatmap indicating the cell confluence over time (% of covered area) corresponding to Fig. 1E. (**C**) Schematic workflow for data transformation to calculate the relative reduction of Torin1-induced ER-phagy flux. Cpd: compounds. (**D**) Relative reduction of ER-phagy flux caused by HIT compounds counteracting Torin1-induced FAM134B- and FAM134C-driven ER-phagy, respectively. HITs were defined as compounds causing >20% relative reduction in ER-phagy flux over a minimum time period of 12h. Source data are provided as source data file.

Supplementary Figure 2

A

FAM134B-HIT list

Selected Hits with >20% Torin-1 induced ERphagy inhibition	Targeted kinase
SGC-CK2-1	Catalytic subunits of CK2: CK2α (CSNK2A1) and CK2α' (CSNK2A2)
VE-822	ATR
KU-55933	ATM
CHIR-124	Chk1
MK-2206 2HCl	Akt1/2/3
SGC-GAK1	GAK
T3-CLK I	CLK
Bisindolylmaleimide I	PKC inhibitor I
NU7441	DNA-PKCs
CHIR-99021	GSK-3α/β

FAM134C-HIT list

Selected Hits with >20% Torin-1 induced ERphagy inhibition	Targeted kinase
SGC-CK2-1	Catalytic subunits of CK2: CK2α (CSNK2A1) and CK2α' (CSNK2A2)
VE-822	ATR
KU-55933	ATM
CHIR-124	Chk1
MK-2206 2HCl	Akt1/2/3
SGC-GAK1	GAK
T3-CLK I	CLK
Bisindolylmaleimide I	PKC inhibitor I
Alisertib	Aurora A
VX-702	p38α MAPK
FRAX486	PAK
LDC0000067	CDK9
RKI-1447	ROCK1 and ROCK2
PZO143	S6K1
Afatinib	EGFR/EGFR2 (HER2/ErbB2)

B

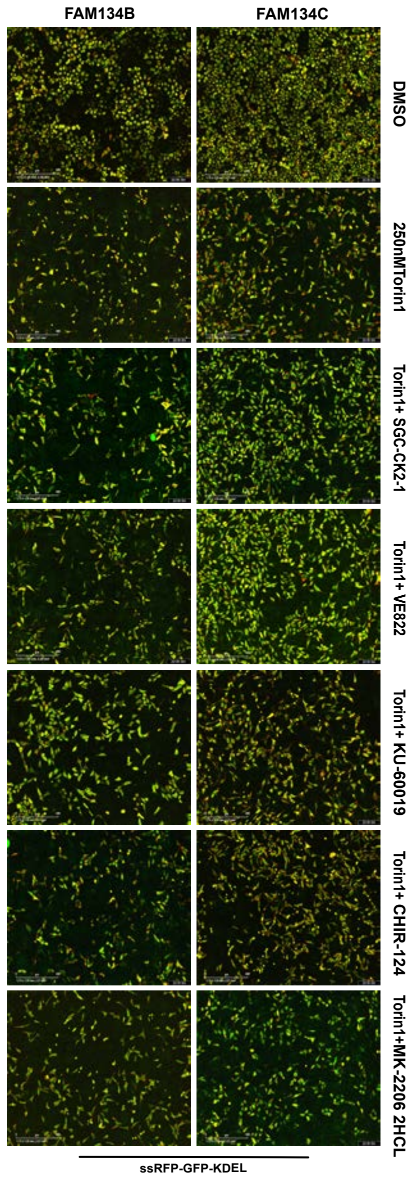

C

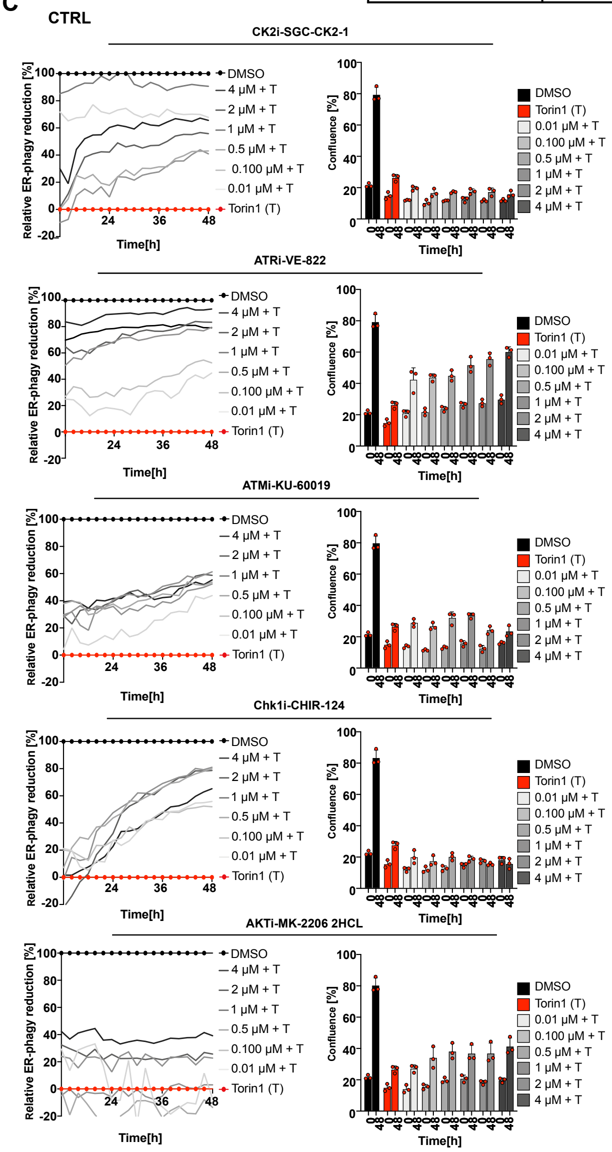

D

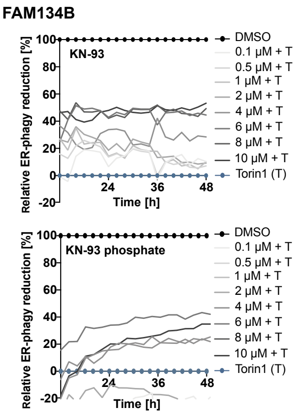

Supplementary figure 2. Validation of chemical kinase screen and identification of CK2 as kinase regulating FAM134B- and FAM134C-dependant ER-phagy

(**A**) List of selected HITs from the ER-phagy screening performed in Fig. 1E showing compounds, and their targets, with an effect on FAM134B (left panel) or FAM134C (right panel) activities. **(B)** Sample pictures of U2OS cells overexpressing FAM134B or FAM134C and stably expressing ssRFP-GFP-KDEL reporter. Pictures were acquired by the IncuCyte® S3 (10x) and show the overlaid GFP and RFP signal from annotated conditions at time point 48h. (**C**) Impact of selected HITs on ER-phagy flux in U2OS control cells. U2OS cells stably expressing KDEL reporter were subjected to a dilution series of selected compound HITs (SGC-CK2-1 (CK2i), VE-822 (ATRi), KU-60019 (ATMi), CHIR-124 (Chk1i) and MK-2206 2HCL (AKTi)) ranging from 0.01 µM to 4 µM combined with Torin1 treatment. Outcome of ER-phagy flux was recorded over 48h. (**D**) Relative reduction of FAM134B-driven ER-phagy flux upon CaMKII inhibition. Source data are provided as source data file.

Data information: Data points shown in **(B-D)** represent the averaged red/green ratio of n=3 images comprising > 100 cells and taken from three independent wells. Data are mean ±SD, [au]: arbitrary unit. Source data are provided as source data file.

Supplementary Figure 3

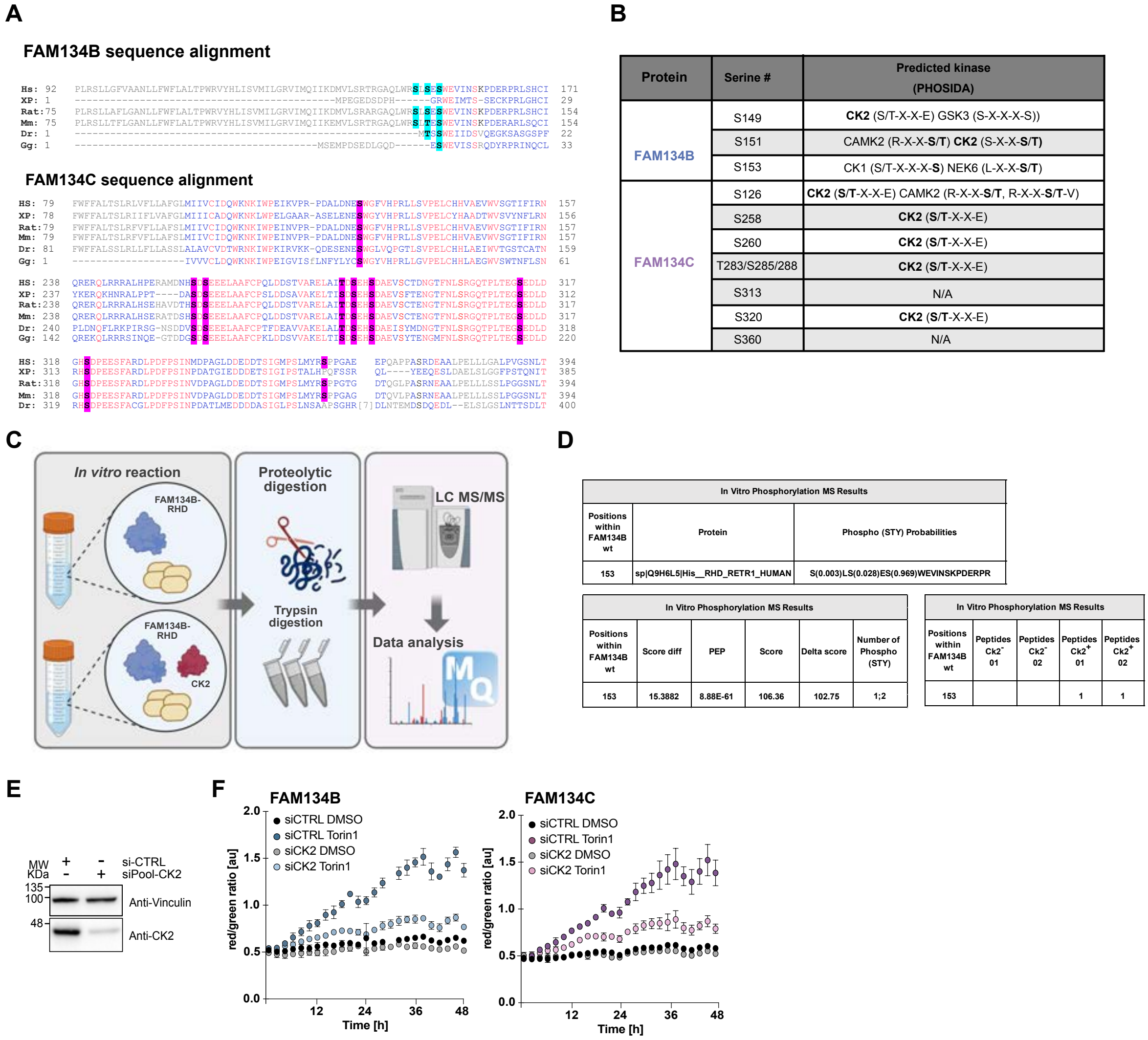

Supplementary figure 3. CK2 mediates FAM134s phosphorylation

(**A**) Multiple sequence alignment of FAM134B and FAM134C showing the conservation level of phosphorylation sites presented in Fig. 3B. (**B**) Predicted kinases phosphorylating identified serines of FAM134B and FAM134C proteins using motif matcher tool PHOSIDA. (**C**) Schematic representation of the workflow of *in vitro* mass spectrometry performed using purified FAM134B RHD domain. **(D)** Tables showing *in vitro* phosphorylation mass spectrometry results. **(E)** Representative western blot image showing KD efficiency of CK2 performed in U2OS cells transfected with control siRNA (siCTRL) or siPOOL targeting CSNK2A1 and CSNK2A3 (siCK2). **(F)** ER-phagy flux in ssRFP-GFP-KDEL U2OS cells overexpressing FAM134B or FAM134C with CK2 present or depleted in the background. ER-phagy flux was assessed either in basal (DMSO) or autophagy-induced (Torin1) conditions.

Total integrated fluorescent intensities (RFP/red and GFP/green) were monitored in the IncuCyte® S3 over a time course of 48 h. Data points represent the averaged red/green ratio of n=3 images comprising >100 cells and taken from three independent wells. Data are mean ±SD, [au]: arbitrary unit. Source data are provided as source data file.

Supplementary Figure 4

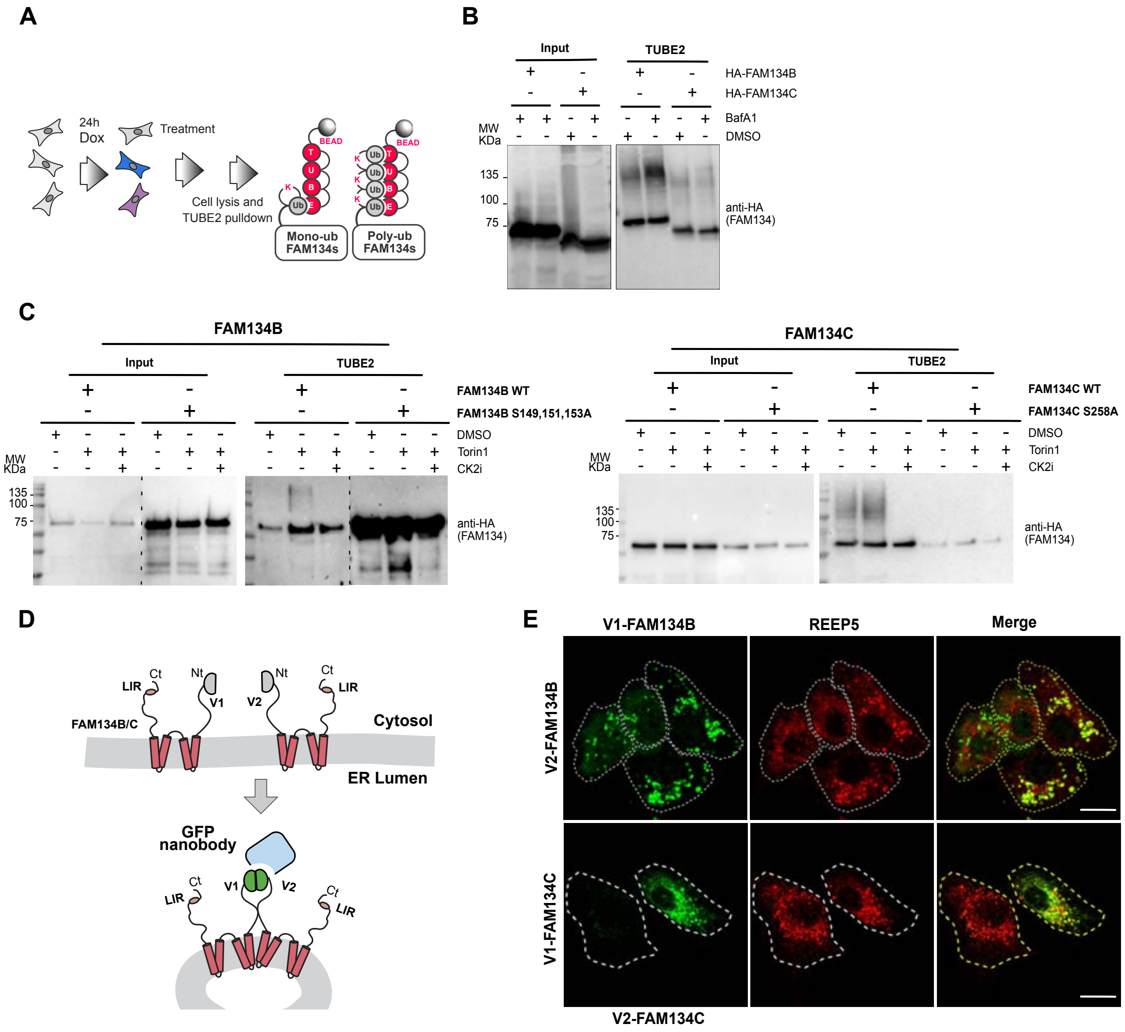

Supplementary figure 4. FAM134B and FAM134C phosphorylation is prerequisite for their ubiquitination and oligomerization in Torin1-dependent manner

(**A**) Schematic representation of Tandem Ubiquitin Binding Entities pulldown assay (TUBE 2) performed in U2OS cells overexpressing FAM134B or FAM134C in DOX-dependent manner which were subjected to different conditions to then purify mono- and poly-ubiquitinated FAM134s proteins. (**B**) Representative western blot image showing enrichment for poly ubiquitinated FAM134 proteins using TUBE 2 assay. U2OS cells overexpressing FAM134B or FAM134C proteins were treated or not with 200 ng/ml BafA1 3 h prior to cell lysis and pulldown experiment. Anti-HA antibody was used to detect HA-tagged FAM134B and FAM134C, as well as ubiquitinated species. n=3 biological replicates**.** **(C)** Representative western blot showing enrichment for poly ubiquitinated FAM134 proteins using TUBE 2 assay. U2OS cells overexpressing FAM134B WT or S149,151,153A, FAM134C WT or S258A

proteins were treated for 6 h with indicated conditions prior to cell lysis. Pulldown of ubiquitinated entities was performed as in Fig. S3A. anti-HA antibody was used to detect HA-tagged FAM134B and FAM134C and ubiquitinated species. n=3 biological replicates. **(D)** Representative scheme outlining the principle behind BiCAP assay using the Venus split system coupled with GFP-Trap pulldown assay. **(E)** Confocal fluorescence microscopy imaging of the BiCAP assay validating the *in cellulo* interaction between V1–FAM134B and V2–FAM134B or V1–FAM134C and V2–FAM134C. Fixed cells expressing the indicated plasmids were stained with REEP5 to mark the ER. Scale bar =10 µm. 10 cells were imaged for control purpose. Source data are provided as source data file.

Supplementary Figure 5

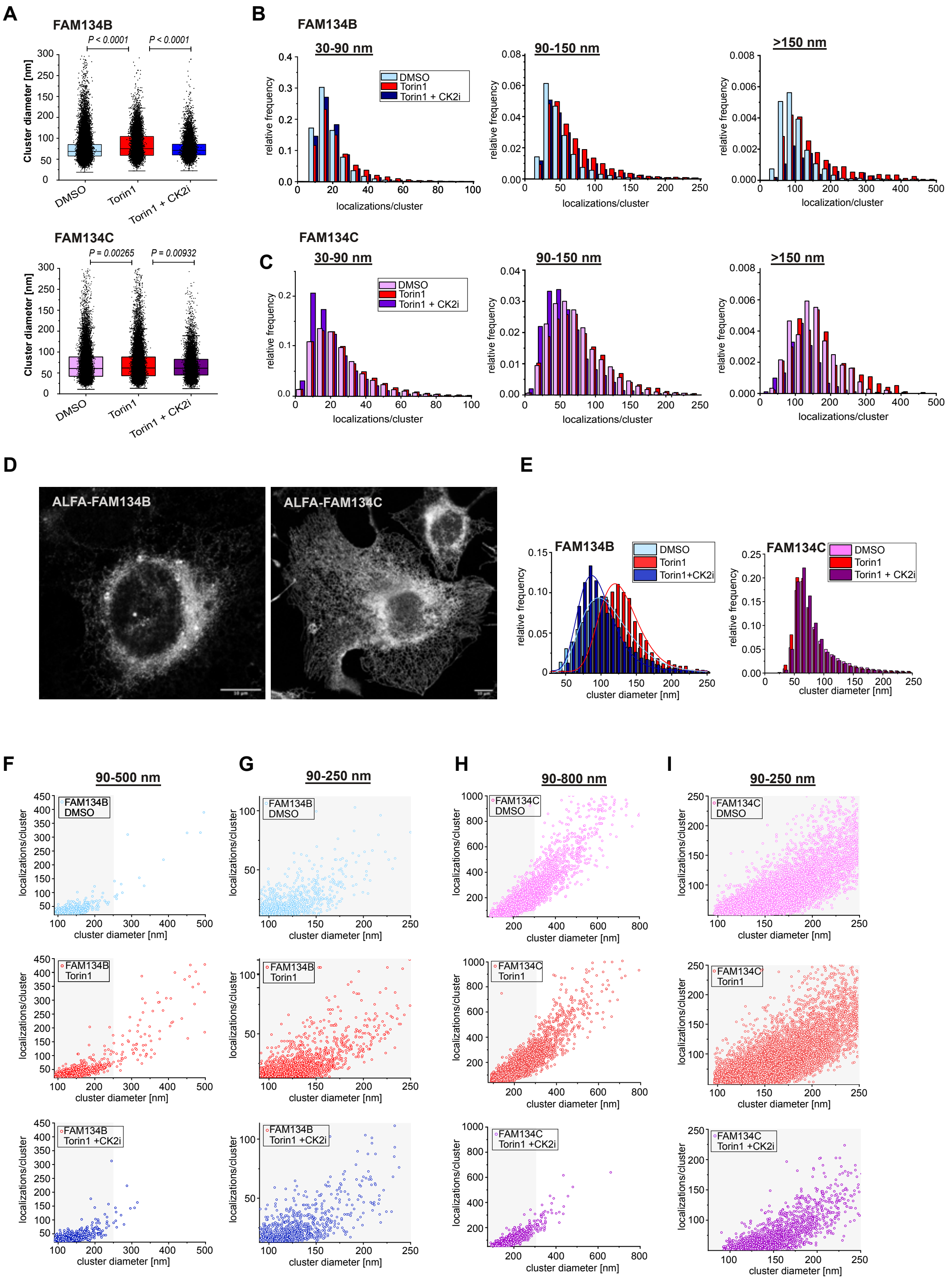

Supplementary figure 5. Phosphorylation of FAM134B and FAM134C regulates cluster size and density

(A) Cluster diameter distributions (boxplots) for FAM134B (upper panel) and FAM134C (lower panel) under basal (DMSO), autophagy induced (Torin1) and Torin1 + CK2i condition. Nanoscale cluster diameters were tested for normal distribution via Shapiro-Wilk normality test and statistical significance via non-parametric Mann-Whitney U-test. For further details see material and methods. (**B-C**) Comparative analysis of FAM134B (A) and FAM134C (B) nanoscale cluster diameters classified into small (30-90 nm), medium (90-150 nm) and large clusters (> 150 nm). **(D)** Super-resolved STED images showing DOX-induced expression (0.1 µg/ml) of ALFA-FAM134B (left panel) and ALFA-FAM134C (right panel) in U2OS cells. Fixed cells were stained with ALFA-tag nanobody to visualize FAM134B/C expression. Both images complement the results of SMLM, where FAM134B is sparsely populated in the ER and FAM134C is densely populated in the ER**. (E)** Quantitative analysis of DNA-PAINT imaging data reports cluster diameters of ALFA-FAM134B (left panel) and ALFA-FAM134C (right panel**)** for DMSO, 250 nM Torin1 and 250 nM Torin1 + 1 µM CK2 inhibitor. Cluster analysis was performed with the DBSCAN clustering algorithm in WT FAM134B- and FAM134C-overexpressing U2OS cells **(F-I).** Comparative analysis of nanoscale clusters densities in ALFA-FAM134B (**F, G**) or ALFA-FAM134C (**H, I**). Fig. S5E-H represent an exact replicate of the experiment conducted in Fig. 5 using ALFA-tag system and show reproducibility in these settings. Source data are provided as source data file.

Supplementary Figure 6

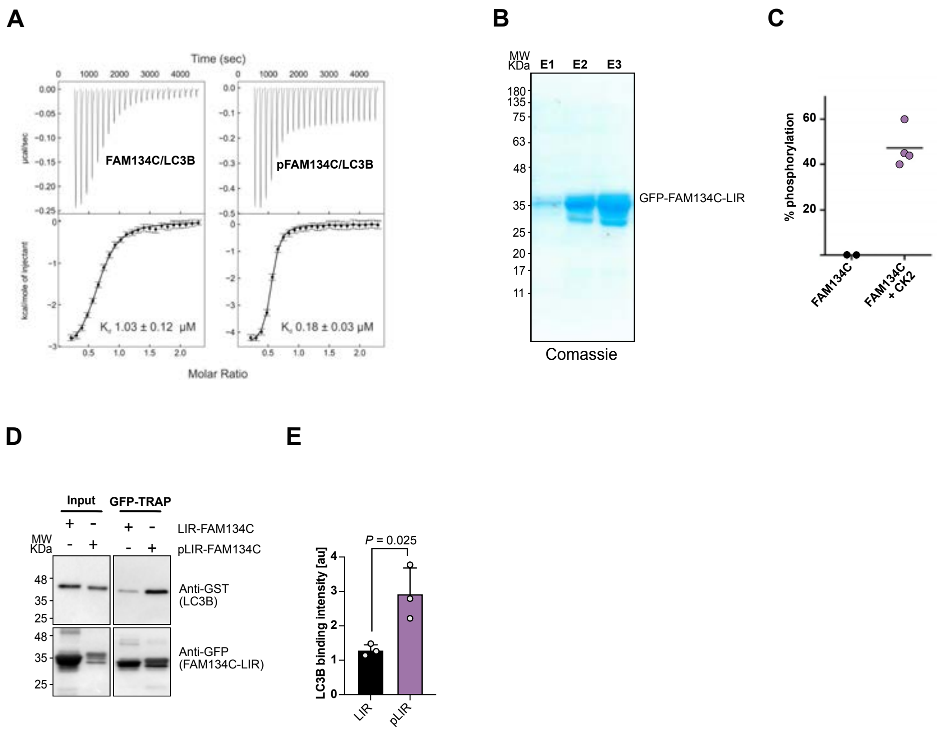

Supplementary figure 6. Phosphorylation of FAM134C increases its affinity for LC3B **(A)** ITC profiles for the titration of purchased FAM134-LIR (left) or FAM13C-pLIR (right) peptide into LC3B. The upper panel shows the raw measurements after baseline correction, the bottom diagram displays the integrated heat per titration step (black circles) with best-fit curves (line). Each measurement was performed in triplicates, representative results are shown. Dissociation constant (K_d) values are indicated, errors represent mean standard deviations. **(B)** SDS-PAGE image showing Coomassie-stained gel of purified GFP-FAM134C-LIR peptide. E1-3 (elution fraction 1-3). **(C)** Phosphorylation level of GFP-FAM134C-LIR in the presence or absence of purified CK2. Peptide phosphorylation was assessed using MALDI (Matric-assisted laser desorption/ionization). **(D,E)** Representative western blot image of GFP-Trap pulldown assay (**D**) and the relative bar plot (**E**) showing binding intensities of phosphorylated or non-phosphorylated GFP-FAM134C-LIR peptide (bait) to GST-LC3B (prey). Anti-GFP was used to detect GFP-FAM134C-LIR while anti-GST antibody was used to detect LC3B. GFP was used as a reference for ratio calculation. Two-sided unpaired student's *t*-test was used to estimate statistical significance. n=3 biological replicates. Data are mean ±SD, [au]: arbitrary unit. Source data are provided as source data file.
