# Supplementary material for: The function of ER-phagy receptors is regulated through phosphorylation-dependent ubiquitination pathways
Source: Nat Commun. 2023 Dec 15;14:8364. doi: 10.1038/s41467-023-44101-5 (PMC10724265; doi:10.1038/s41467-023-44101-5)
Supplement: Supplementary file 3 — Reporting Summary [file 41467_2023_44101_MOESM3_ESM.pdf]

nature portfolio

Corresponding author(s): Alexandra Stolz
Last updated by author(s): Nov 16, 2023

Reporting Summary

Nature Portfolio wishes to improve the reproducibility of the work that we publish. This form provides structure for consistency and transparency in reporting. For further information on Nature Portfolio policies, see our Editorial Policies and the Editorial Policy Checklist.

Statistics

For all statistical analyses, confirm that the following items are present in the figure legend, table legend, main text, or Methods section.

n/a	Confirmed	
☐	☒	The exact sample size (n) for each experimental group/condition, given as a discrete number and unit of measurement
☐	☒	A statement on whether measurements were taken from distinct samples or whether the same sample was measured repeatedly
☐	☒	The statistical test(s) used AND whether they are one- or two-sided<br>*Only common tests should be described solely by name; describe more complex techniques in the Methods section.*
☒	☐	A description of all covariates tested
☒	☐	A description of any assumptions or corrections, such as tests of normality and adjustment for multiple comparisons
☐	☒	A full description of the statistical parameters including central tendency (e.g. means) or other basic estimates (e.g. regression coefficient) AND variation (e.g. standard deviation) or associated estimates of uncertainty (e.g. confidence intervals)
☐	☒	For null hypothesis testing, the test statistic (e.g. F, t, r) with confidence intervals, effect sizes, degrees of freedom and P value noted<br>*Give P values as exact values whenever suitable.*
☒	☐	For Bayesian analysis, information on the choice of priors and Markov chain Monte Carlo settings
☒	☐	For hierarchical and complex designs, identification of the appropriate level for tests and full reporting of outcomes
☒	☐	Estimates of effect sizes (e.g. Cohen's d, Pearson's r), indicating how they were calculated

Our web collection on statistics for biologists contains articles on many of the points above.

Software and code

Policy information about availability of computer code

Data collection	Micro-Manager 1.4.22, IncuCyte S3 2020B (essenbioscience), NIS Elements (Nikon, Japan), LCControl (Agilent, USA), Image Lab 6.1 (BioRad).
Data analysis	ImageLab 6.1 (BioRad), Affinity Designer 1.10.4., Prism 8, Picasso software 0.6.1. (https://github.com/jungmannlab/picasso), ViSP 1.0 (https://doi.org/10.1038/nmeth.2566), Origin 2019, ImageJ 1.53n, Python 3.8., IncuCyte S3 2020B (essenbioscience), MaxQuant (v 2.1.0.0).

For manuscripts utilizing custom algorithms or software that are central to the research but not yet described in published literature, software must be made available to editors and reviewers. We strongly encourage code deposition in a community repository (e.g. GitHub). See the Nature Portfolio guidelines for submitting code & software for further information.

Data

Policy information about availability of data

All manuscripts must include a data availability statement. This statement should provide the following information, where applicable:

- Accession codes, unique identifiers, or web links for publicly available datasets
- A description of any restrictions on data availability
- For clinical datasets or third party data, please ensure that the statement adheres to our policy

The mass spectrometry proteomics data have been deposited to the proteomeXchange Consortium via the PRIDE partner repository with the dataset identifier PXD043003 [https://www.ebi.ac.uk/pride/archive/projects/PXD043003]. Source data file containing MS/MS spectra, all uncropped gels and raw/processed raw data

for all graphs presented in the figures is provided with this publication as 'source data' file.

Research involving human participants, their data, or biological material

Policy information about studies with human participants or human data. See also policy information about sex, gender (identity/presentation), and sexual orientation and race, ethnicity and racism.

Reporting on sex and gender	The manuscript does not involve human research participants
Reporting on race, ethnicity, or other socially relevant groupings	The manuscript does not involve human research participants
Population characteristics	The manuscript does not involve human research participants
Recruitment	The manuscript does not involve human research participants
Ethics oversight	The manuscript does not involve human research participants

Note that full information on the approval of the study protocol must also be provided in the manuscript.

Field-specific reporting

Please select the one below that is the best fit for your research. If you are not sure, read the appropriate sections before making your selection.

[x] Life sciences [ ] Behavioural & social sciences [ ] Ecological, evolutionary & environmental sciences

For a reference copy of the document with all sections, see nature.com/documents/nr-reporting-summary-flat.pdf

Life sciences study design

All studies must disclose on these points even when the disclosure is negative.

Sample size	No prior sample size calculation was performed. All experiments were performed at least three times. The respective details are mentioned in figure legends.<br>For ER-phagy flux data, sample size was determined based on similar study: e.g. Reggio A, et. al. (2021) Role of FAM134paralogues in endoplasmic reticulum remodeling, ER-phagy, and Collagen quality control. EMBOR 22:e52289 and included >100 cells per biological replicate.<br>For super resolution microscopy experiments, sample size was determined based on similar study in the field: e.g. Gonzáles A, et. al. (2023) Ubiquitination regulates ER-phagy and remodelling of endoplasmic reticulum. Nature 618, pages394–401.
Data exclusions	For ER-phagy flux assays individual wells of technical replicates have been removed in case there was a foreign objects, large bubbles, or similar events obviously disturbing fluorescence analysis within a specific well, resulting in less than tree technical replicates (1 or two) per biological replicate. No other data was excluded from analysis.
Replication	All data with statistical analysis have been repeated at least 3 times with consistent, similar results. Super resolution microscopy experiments were repeated at least 3 times in different systems using different tools to ensure reproducibility.
Randomization	No randomization was necessary. Images were automatically acquired for live cell imaging or super resolution microscopy. For Mass spectrometry or biochemical assays, same treatment were applied to facilitate comparisons across various conditions
Blinding	N/A: No blinding was performed due to the nature of the investigation.

Reporting for specific materials, systems and methods

We require information from authors about some types of materials, experimental systems and methods used in many studies. Here, indicate whether each material, system or method listed is relevant to your study. If you are not sure if a list item applies to your research, read the appropriate section before selecting a response.

Materials & experimental systems

n/a	Involved in the study
☐	☒ Antibodies
☐	☒ Eukaryotic cell lines
☒	☐ Palaeontology and archaeology
☒	☐ Animals and other organisms
☒	☐ Clinical data
☒	☐ Dual use research of concern
☒	☐ Plants

Methods

n/a	Involved in the study
☒	☐ ChIP-seq
☒	☐ Flow cytometry
☒	☐ MRI-based neuroimaging

Antibodies

Antibodies used

Primary antibodies used in this study are listed below:
-Rabbit anti-HA tag (Provider: Cell signalling, Cat# 3724T). Dilution: 1/2000 for immunoblotting.
-Rat anti-HA tag (Provider: Roche, Cat# 11867432001). Dilution: 1/5000 for immunofluorescence.
-Rabbit anti-FAM134B (Provider: Sigma Prestige, Cat# HPA012077). Dilution: 1/1000 for immunoblotting.
-Rabbit anti-FAM134C (Provider: Sigma Prestige, Cat# HPA016492). Dilution: 1/1000 for immunoblotting.
-Mouse anti-REEP5 (Provider: Santa Cruz Biotechnology, Cat# sc-393508). Dilution: 1/1000 for immunofluorescence.
-Rabbit anti-GST (Provider: Cell Signaling Technology, Cat# 2625S). Dilution: 1/2000 for immunoblotting.
-Mouse anti-GFP (Provider: Santa Cruz Biotechnology, Cat# sc-9996). Dilution: 1/2000 for immunoblotting.
-Rabbit anti-CK2 (Provider: Cell signaling Technology, Cat# 2656S). Dilution: 1/1000 for immunoblotting.
-Mouse anti-ubiquitin PD4 (Provider: abcam, Cat# ab7254). Dilution: 1/1000 for immunoblotting.
-Rabbit anti-GAPDH (Provider: Cell signalling, Cat# 2118L).Dilution: 1/1000 for immunoblotting.

Following secondary antibodies were used at 1/10000 dilution for immunoblotting:
-Anti-mouse (Provider: Thermo Fisher Scientific, Cat# 31326).
-Anti-rabbit (Provider: Thermo Fisher Scientific, Cat# 32460)
-Anti-rat (Provider: Cell signalling, Cat# 70775)

Following secondary antibodies were used at 1/500 dilution for immunofluorescent staining and for super-resolution microscopy:
-Anti-mouse Alexa Fluor® 532 (Provider: Life technologies, Cat# A11002)
-Anti-rabbit (Provider: AffiniPure, Cat# 711-005-152)
-Anti-mouse (Provider: AffiniPure, Cat# 115-005-003)
both antibodies are covalently labeled with short oligonucleotide strands anti-P1 (5´-ATCTACATATT-3´) and anti-P5 (5´-TATGTAACTTT-3´), respectively.

Validation

-Rabbit anti-HA tag (Provider: Cell signalling, Cat# 3724T)
Application: Western Blotting, Immunoprecipitation, Immunohistochemistry (Paraffin),Immunofluorescence (Immunocytochemistry), Flow Cytometry (Fixed/Permeabilized), Chromatin IP
Validation statement can be found at manufacturer's website: https://www.cellsignal.com/products/primary-antibodies/ha-tag-c29f4-rabbit-mab/3724?_requestid=7270334

-Rat anti-HA tag (Provider: Roche, Cat# 11867432001)
Application:Dot blots, ELISA, Immunocytochemistry, Immunoprecipitation, Western blot
Validation statement can be found at manufacturer's website: https://www.sigmaaldrich.com/DE/en/product/roche/roahaha

-Rabbit anti-FAM134B (Provider: Sigma Prestige, Cat# HPA012077)
Application: Immunofluorescence, Immunoprecipitation, Western blot
Validation statement: All Prestige Antibodies Powered by Atlas Antibodies are developed and validated by the Human Protein Atlas (HPA) project and as a result, are supported by the most extensive characterization in the industry. https://www.sigmaaldrich.com/DE/en/product/sigma/hpa012077

-Rabbit anti-FAM134C (Provider: Sigma Prestige, Cat# HPA016492).
Application: Immunocytochemistry, Immunofluorescence, Immunoprecipitation, Western blot.
Validation statement: All Prestige Antibodies Powered by Atlas Antibodies are developed and validated by the Human Protein Atlas (HPA) project and as a result, are supported by the most extensive characterization in the industry. https://www.sigmaaldrich.com/DE/en/product/sigma/hpa016492

-Mouse anti-REEP5 (Provider: Santa Cruz Biotechnology, Cat# sc-393508)
Application: Immunocytochemistry, Immunofluorescence, Western blot.
Validation statement can be found at manufacturer's website: https://www.scbt.com/p/reep5-antibody-h-10

-Rabbit anti-GST (Provider: Cell Signaling Technology, Cat# 2625S)
Application:Western Blot, Immunoprecipitation, Immunohistochemistry ChIP-Chromatin Immunoprecipitation, Dot Blot,

Immunofluorescence, Flow Cytometry
Validation statement can be found at manufacturer's website: https://www.cellsignal.com/products/primary-antibodies/gst-91g1-rabbit-mab/2625

-Mouse anti-GFP (Provider: Santa Cruz Biotechnology, Cat# sc-9996)
Application: Immunofluorescence, Immunoprecipitation, Western blot.
Validation statement can be found at manufacturer's website: https://www.scbt.com/p/gfp-antibody-b-2

-Rabbit anti-CK2 (Provider: Cell signaling Technology, Cat# 2656S).
Application:Western Blot, Immunoprecipitation, Immunohistochemistry ChIP-Chromatin Immunoprecipitation, Dot Blot, Immunofluorescence, Flow Cytometry.
Validation statement can be found at manufacturer's website: https://www.cellsignal.com/products/primary-antibodies/ck2a-antibody/2656

-Mouse anti-ubiquitin PD4 (Provider: abcam, Cat# ab7254).
Application: Immunocytochemistry, Western blot.
Validation statement can be found at manufacturer's website: https://www.abcam.com/products/primary-antibodies/ubiquitin-antibody-ubi-1-ab7254.html

-Rabbit anti-GAPDH (Provider: Cell signalling, Cat# 2118L)
Application: Western Blot, Immunohistochemistry (Paraffin),Immunofluorescence (Immunocytochemistry), Flow Cytometry (Fixed/Permeabilized),.
Validation statement can be found at manufacturer's website: https://www.cellsignal.com/products/primary-antibodies/gapdh-14c10-rabbit-mab/2118

-Anti-mouse (Provider: Thermo Fisher Scientific, Cat# 31326).
Application:Western Blot, Immunoprecipitation
Validation statement can be found at manufacturer's website: https://www.thermofisher.com/antibody/product/Goat-anti-Mouse-IgM-Mu-chain-Secondary-Antibody-Polyclonal/31326
-Anti-rabbit (Provider: Thermo Fisher Scientific, Cat# 32460)
Application:Western Blot, IImmunohistochemistry, ELISA.
Validation statement can be found at manufacturer's website: https://www.thermofisher.com/antibody/product/Goat-anti-Rabbit-IgG-H-L-Secondary-Antibody-Polyclonal/32460

-Anti-rat (Provider: Cell signalling, Cat# 70775)
Application:Western Blot, Immunoprecipitation, Immunohistochemistry, Dot Blot, Immunofluorescence, Flow Cytometry.
Validation statement can be found at manufacturer's website: https://www.cellsignal.com/products/secondary-antibodies/anti-rat-igg-hrp-linked-antibody/7077

-Anti-mouse Alexa Fluor® 532 (Provider: Life technologies, Cat# A11002)
Application:Immunohistochemistry, Immunofluorescence, Flow Cytometry.
Validation statement can be found at manufacturer's website: https://www.thermofisher.com/antibody/product/Goat-anti-Mouse-IgG-H-L-Cross-Adsorbed-Secondary-Antibody-Polyclonal/A-11002

-Anti-rabbit (Provider: AffiniPure, Cat# 711-005-152)
Application: Western Blot, Immunoprecipitation, Immunohistochemistry, Immunofluorescence, Flow Cytometry.
Validation statement can be found at manufacturer's website: https://www.jacksonimmuno.com/catalog/products/711-005-152

-Anti-mouse (Provider: AffiniPure, Cat# 115-005-003)
Application: Western Blot, Immunoprecipitation, Immunohistochemistry, Immunofluorescence, Flow Cytometry.
Validation statement can be found at manufacturer's website: https://www.jacksonimmuno.com/catalog/products/115-005-003

Eukaryotic cell lines

Policy information about cell lines and Sex and Gender in Research

Cell line source(s)	-HEK293T was purchased from ATCC (CRL-3216)<br>-U2OS Trex cells were a gift from Stephen Blacklow (Brigham and Women's Hospital and Harvard Medical School)<br>-MEFs were provided by Prof. Christian Huebner (Jena University)
Authentication	Cell lines authentication by morphology was performed using microscopy.
Mycoplasma contamination	Cell lines were tested negative for Mycoplasma contamination.
Commonly misidentified lines (See ICLAC register)	No commonly misidentified cell lines were used.
