# Supplementary material for: The function of ER-phagy receptors is regulated through phosphorylation-dependent ubiquitination pathways
Source: Nat Commun. 2023 Dec 15;14:8364. doi: 10.1038/s41467-023-44101-5 (PMC10724265; doi:10.1038/s41467-023-44101-5)

FAM134B-S149

Raw File	Scan	Method	Score	m/z	Gene names
20220311_MAA_PS_FAMB_DMSO_4	20569	FTMS; HCD	147.67	737.35	FAM134B

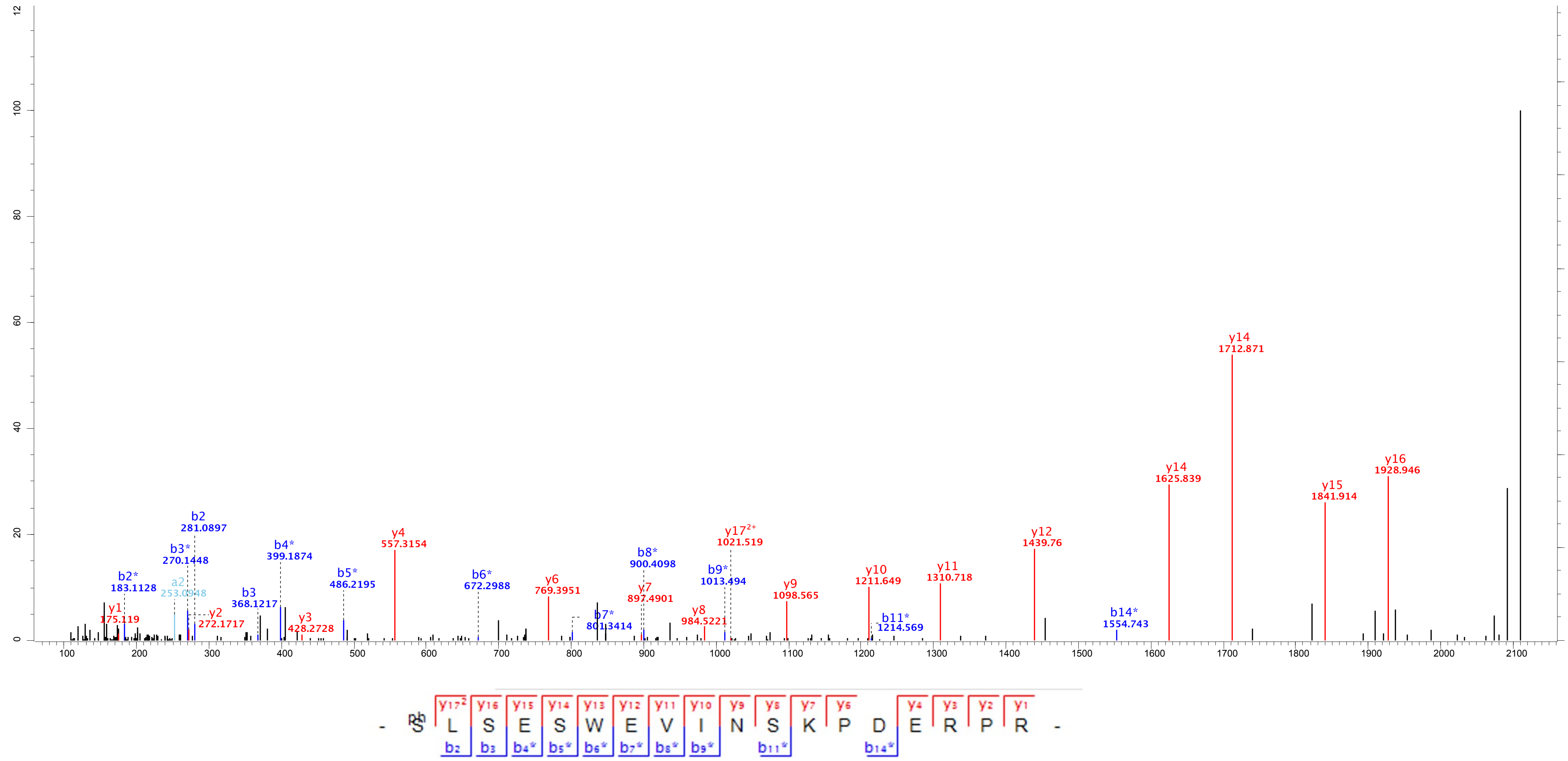

FAM134B-S149 Zoom in lower ions

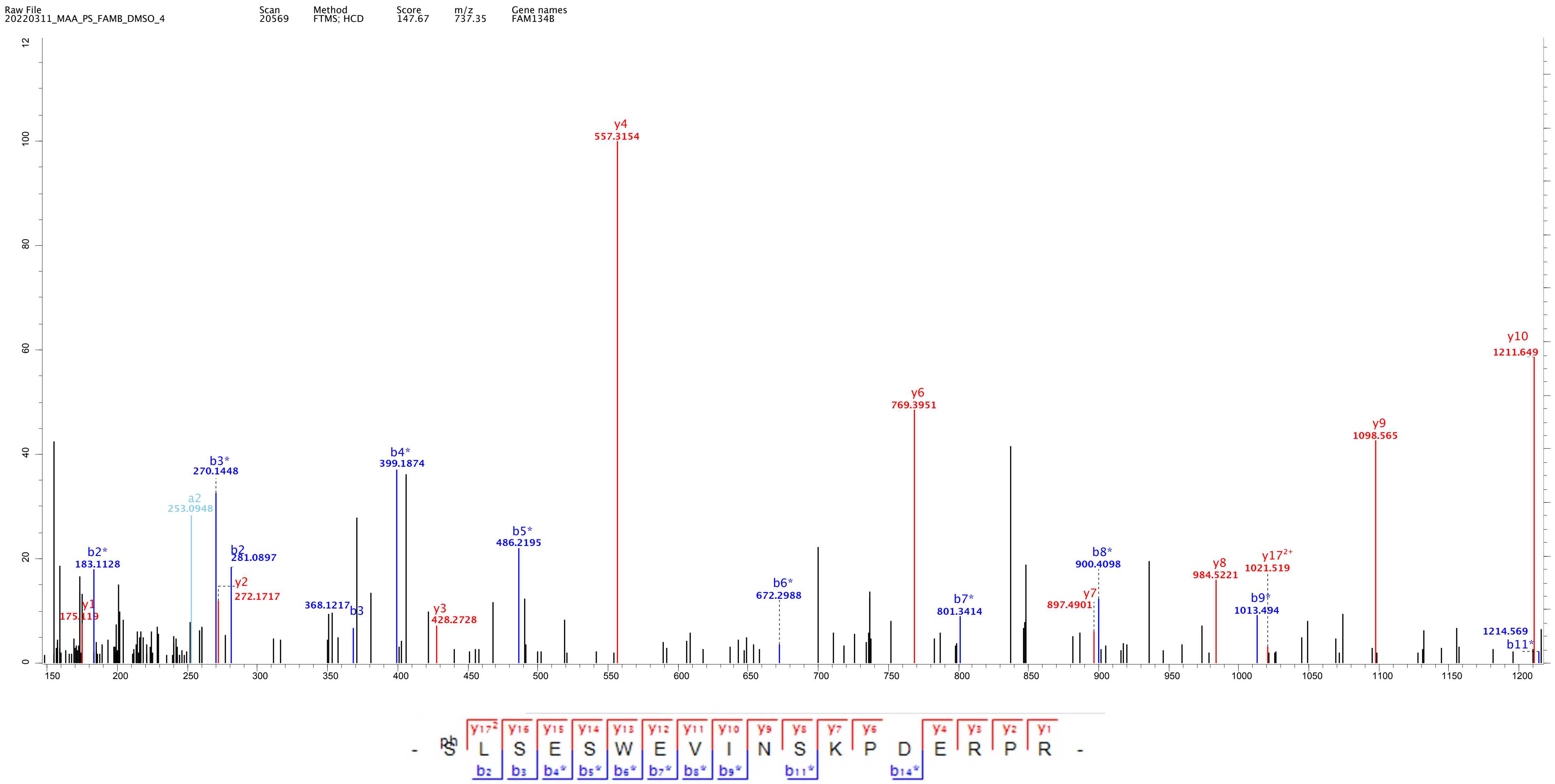

FAM134B-S151

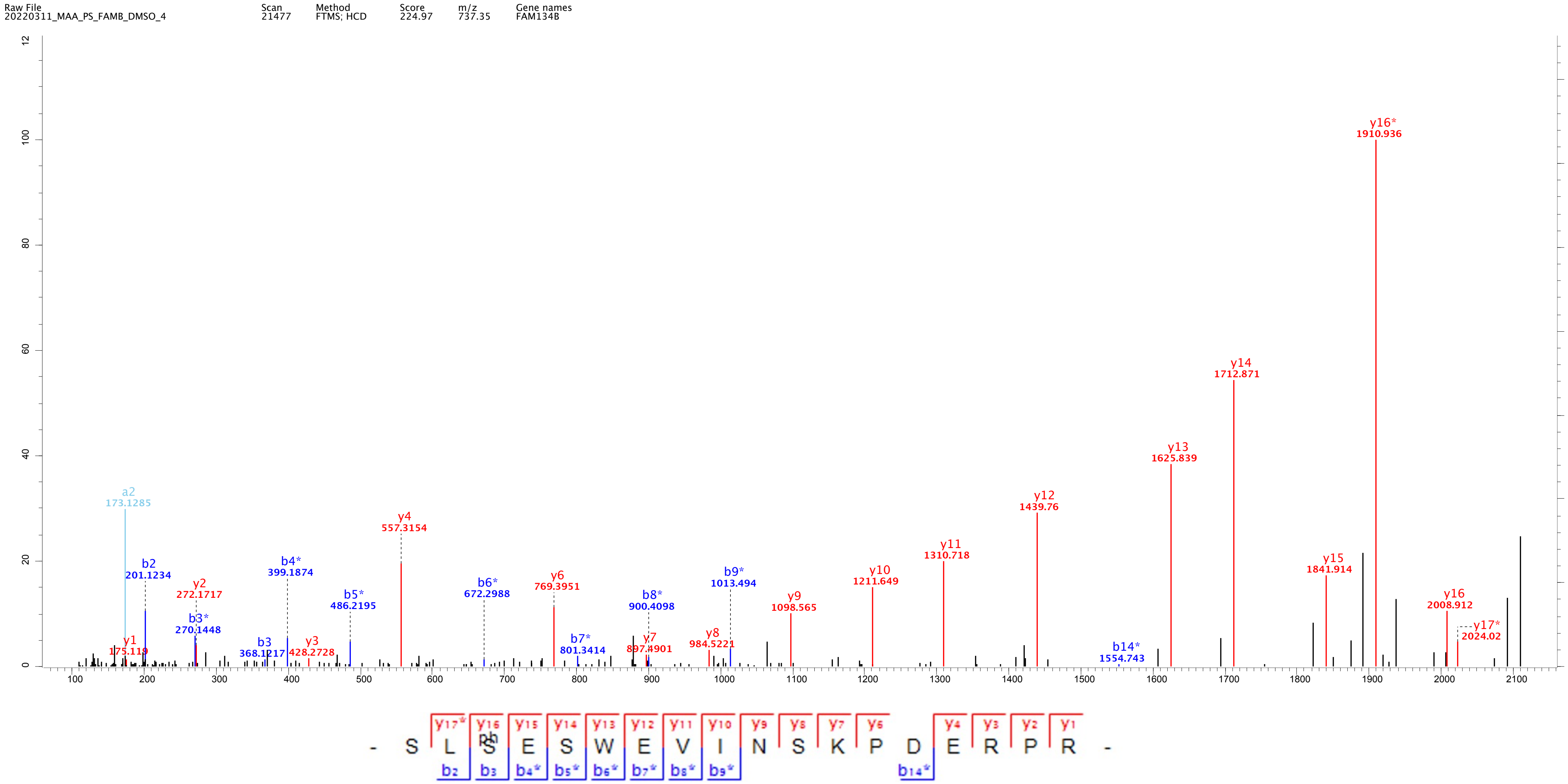

FAM134B-S151 Zoom in lower ions

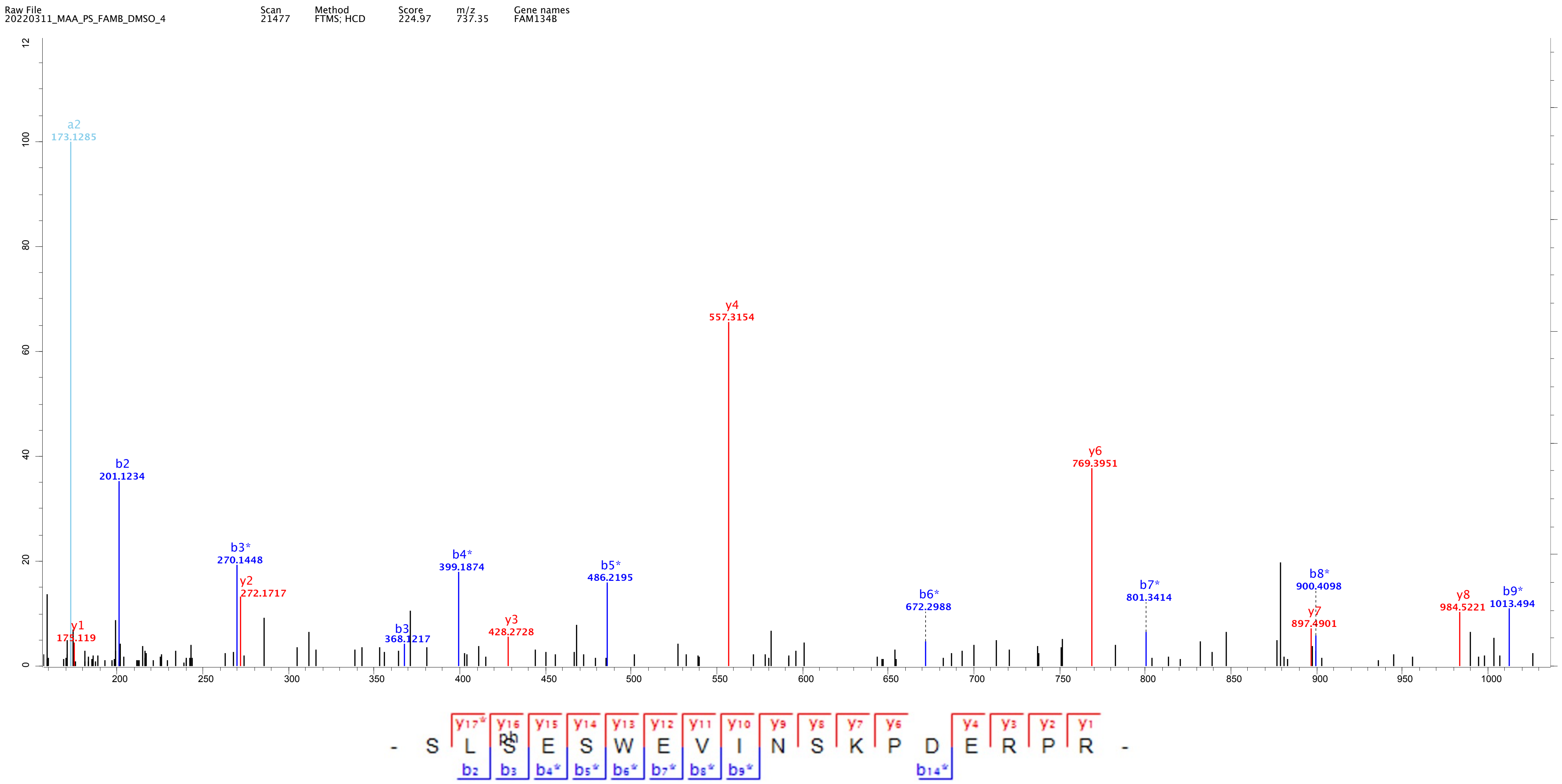

FAM134B-S153

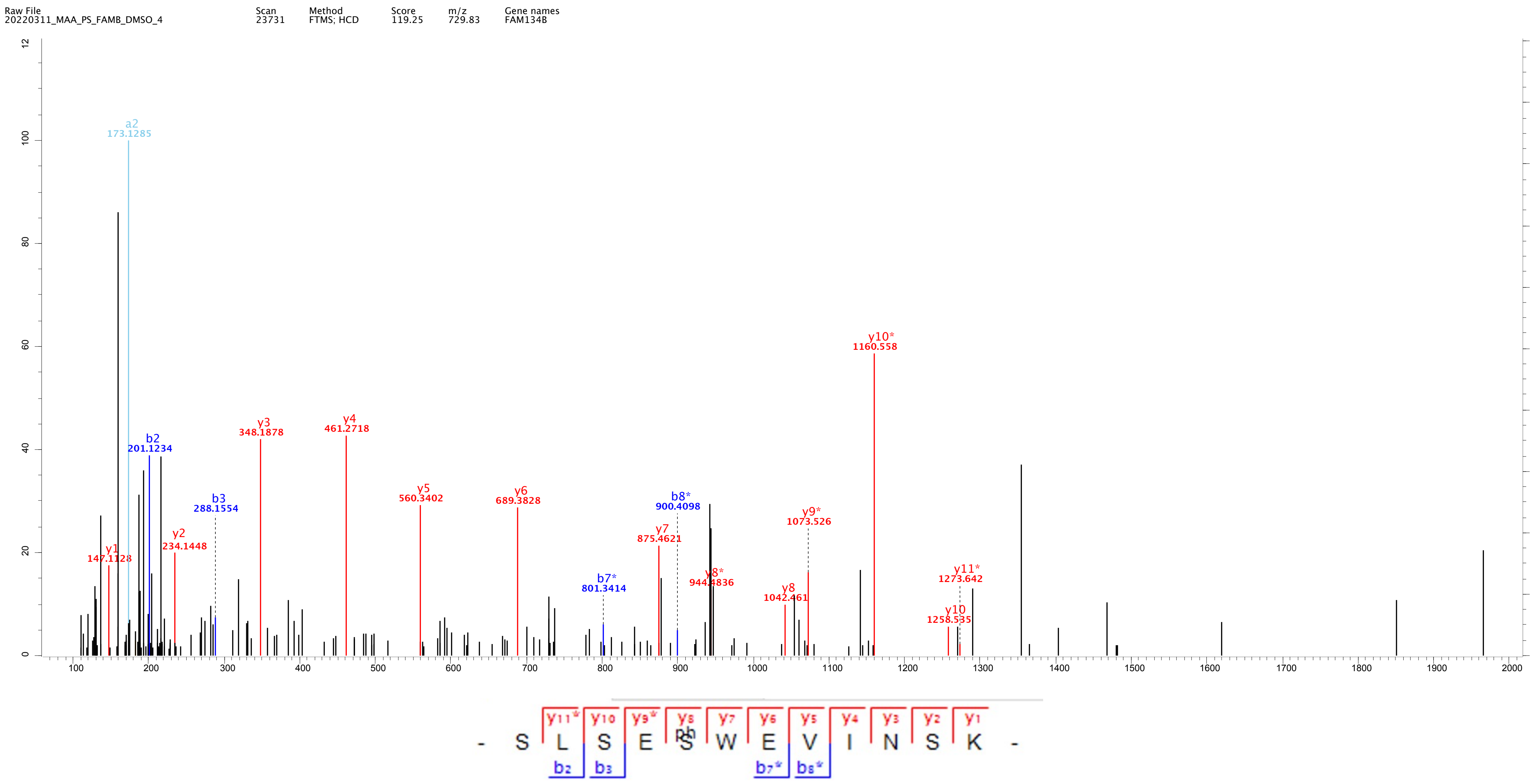

FAM134B-S153 Zoom in lower ions

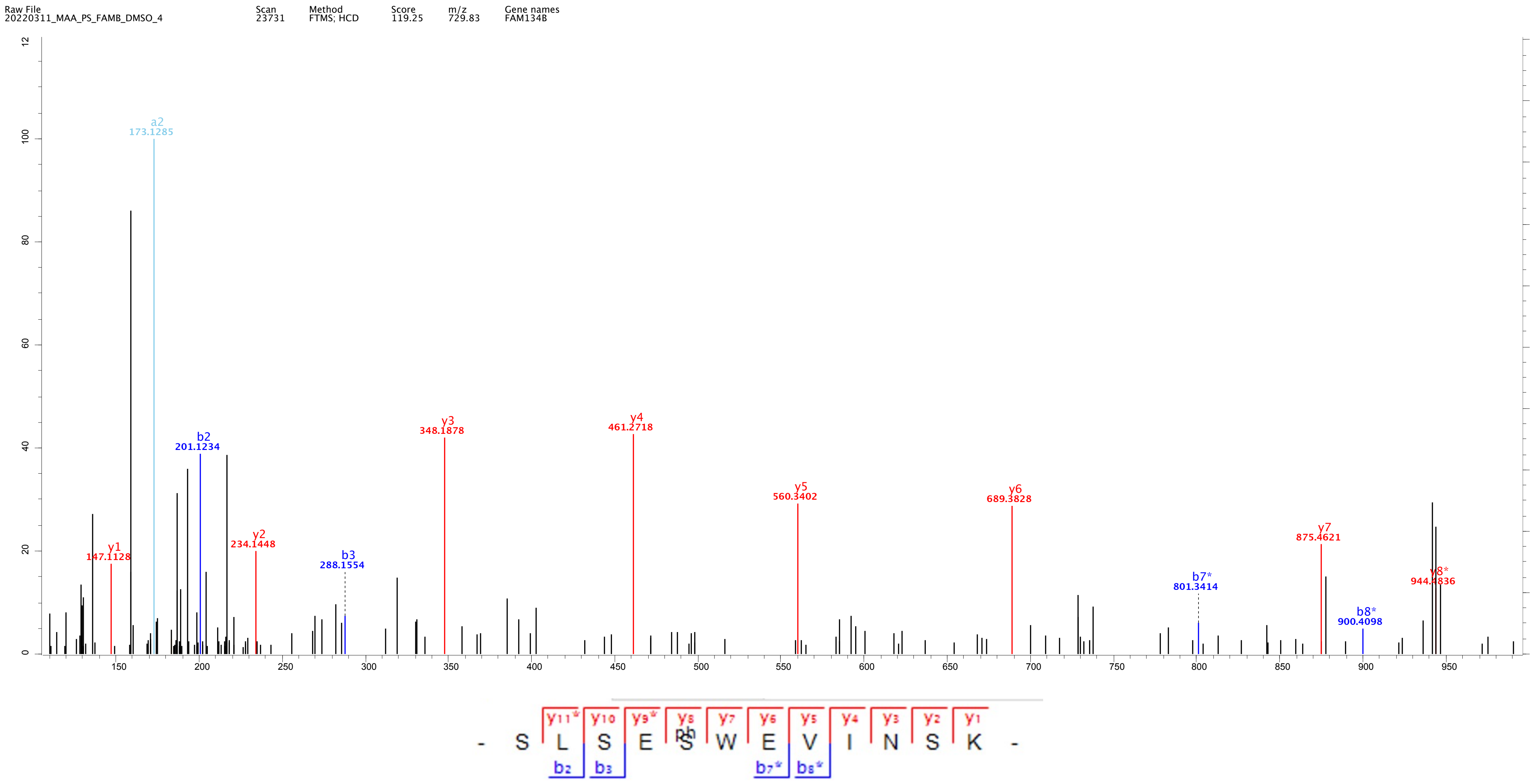

FAM134B-S153 Zoom in Y8*

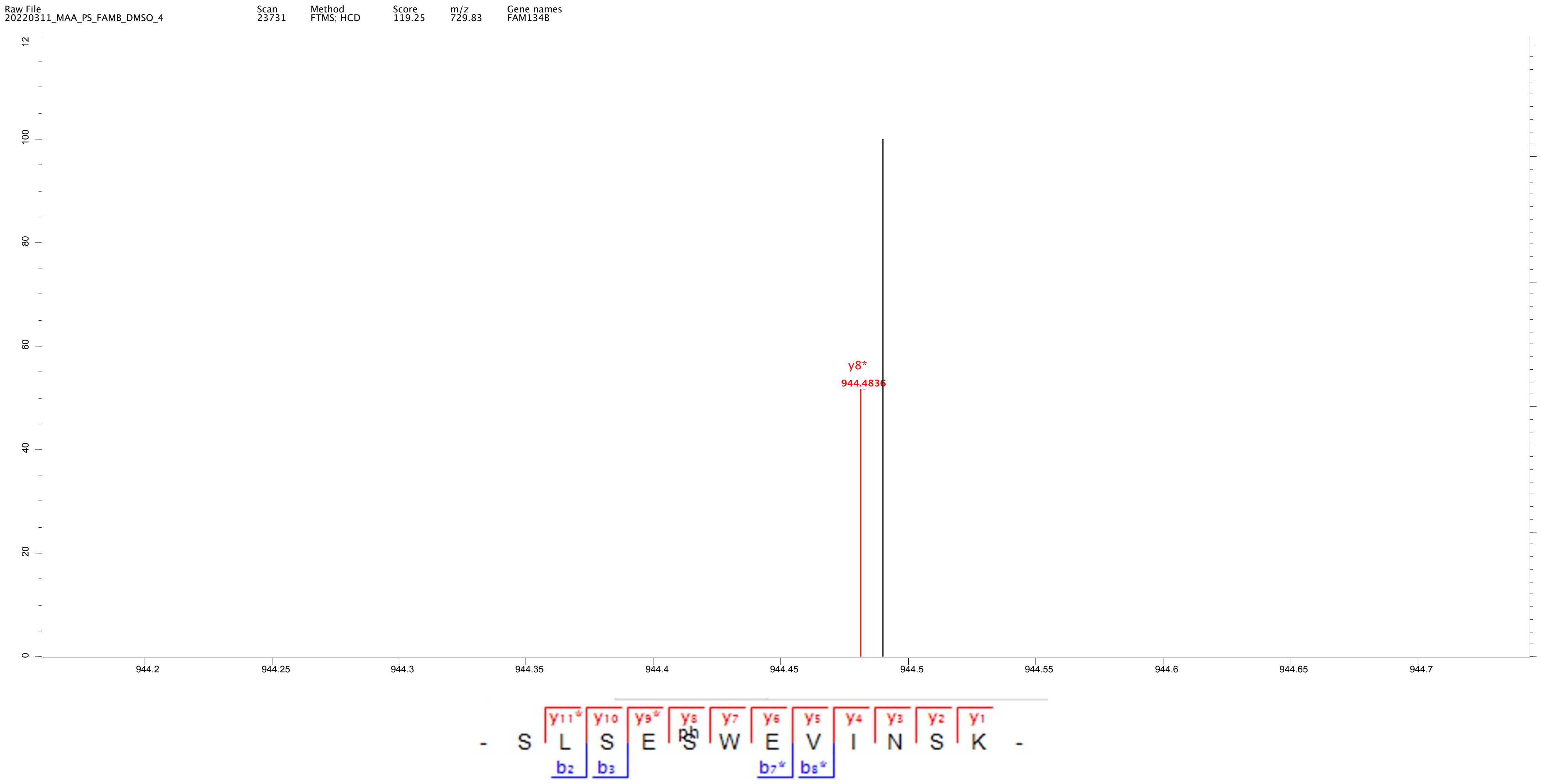

FAM134C-S258

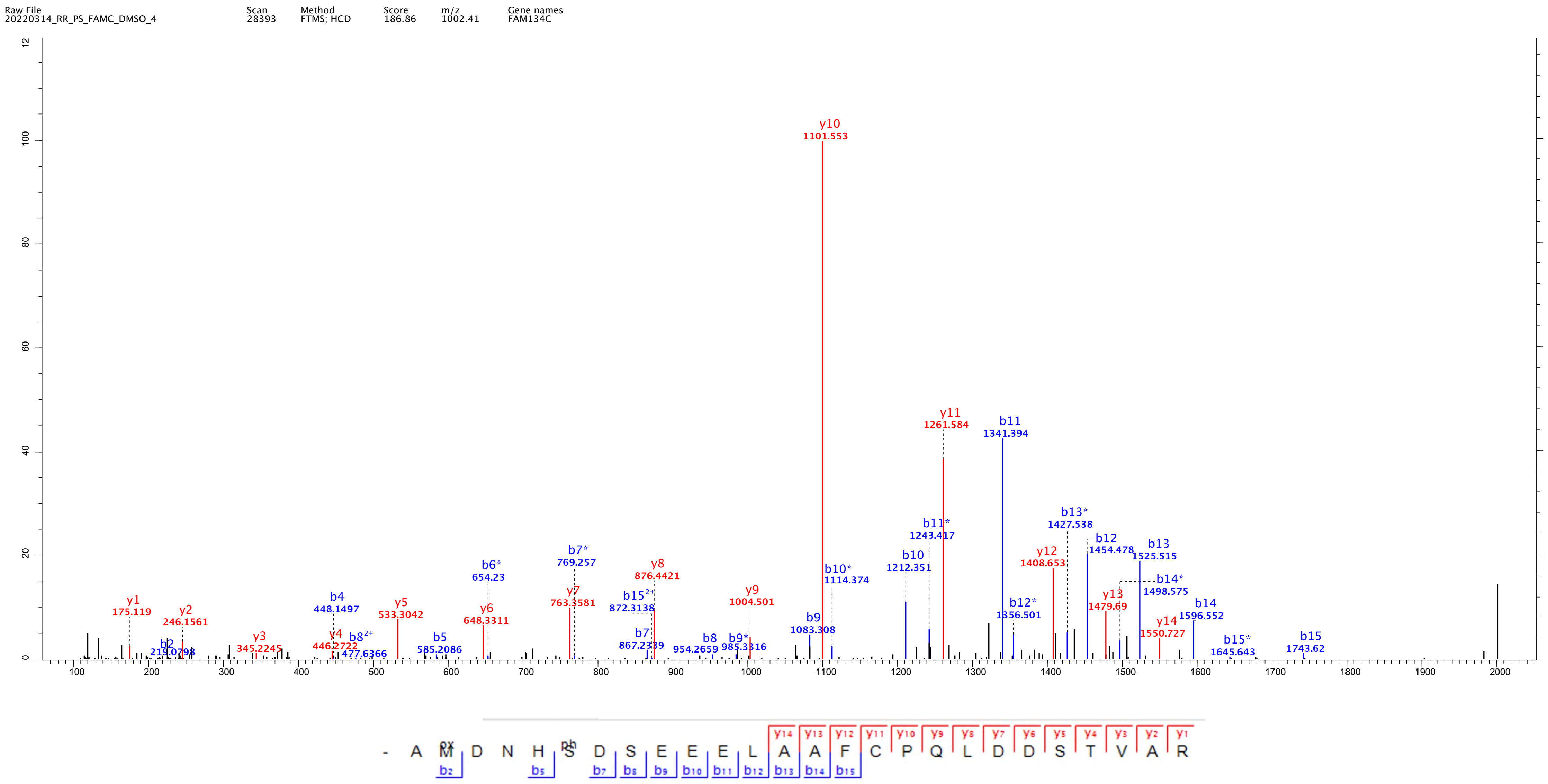

FAM134C-S258- Low ions

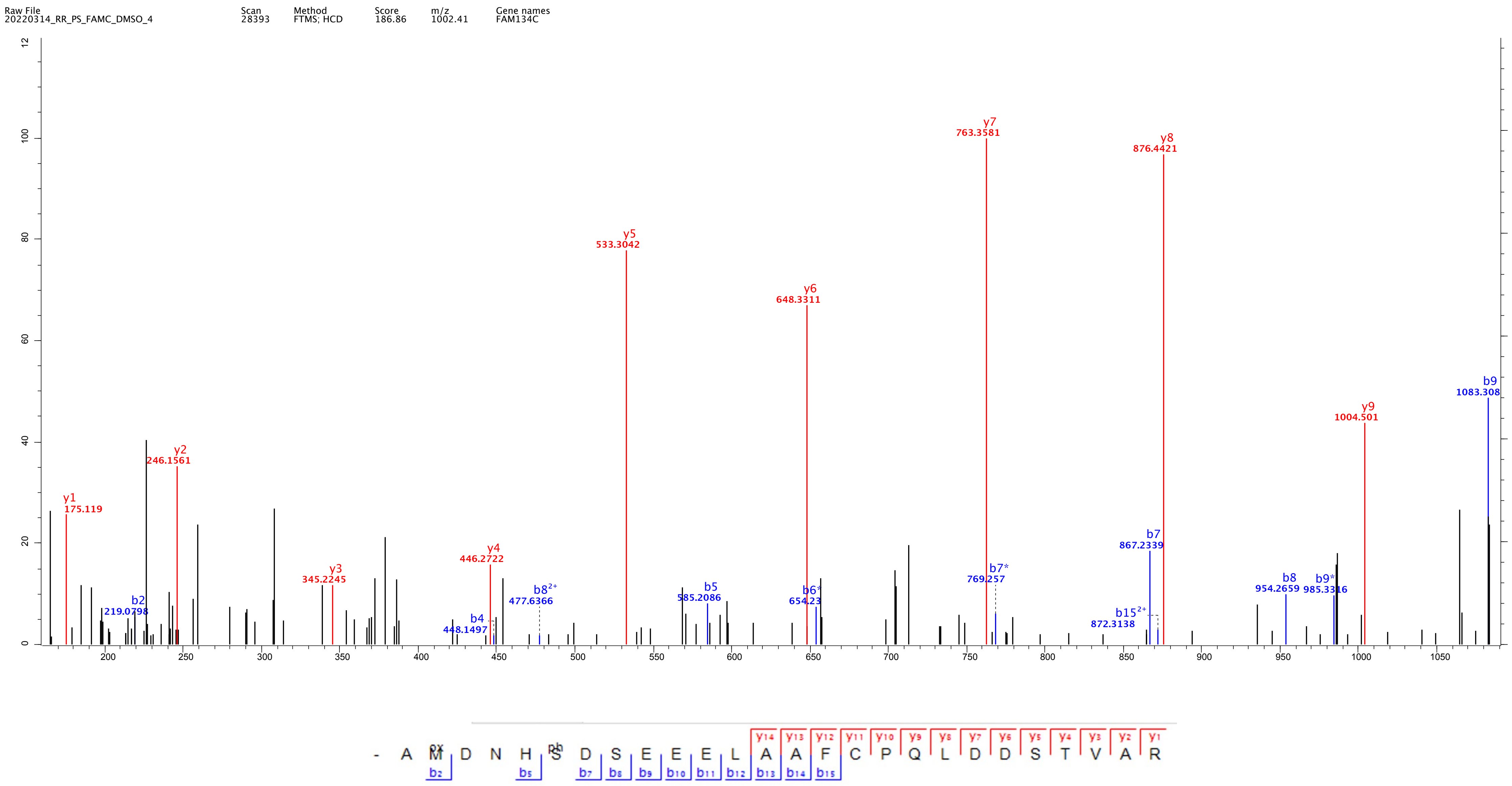

FAM134C-S258- High ions

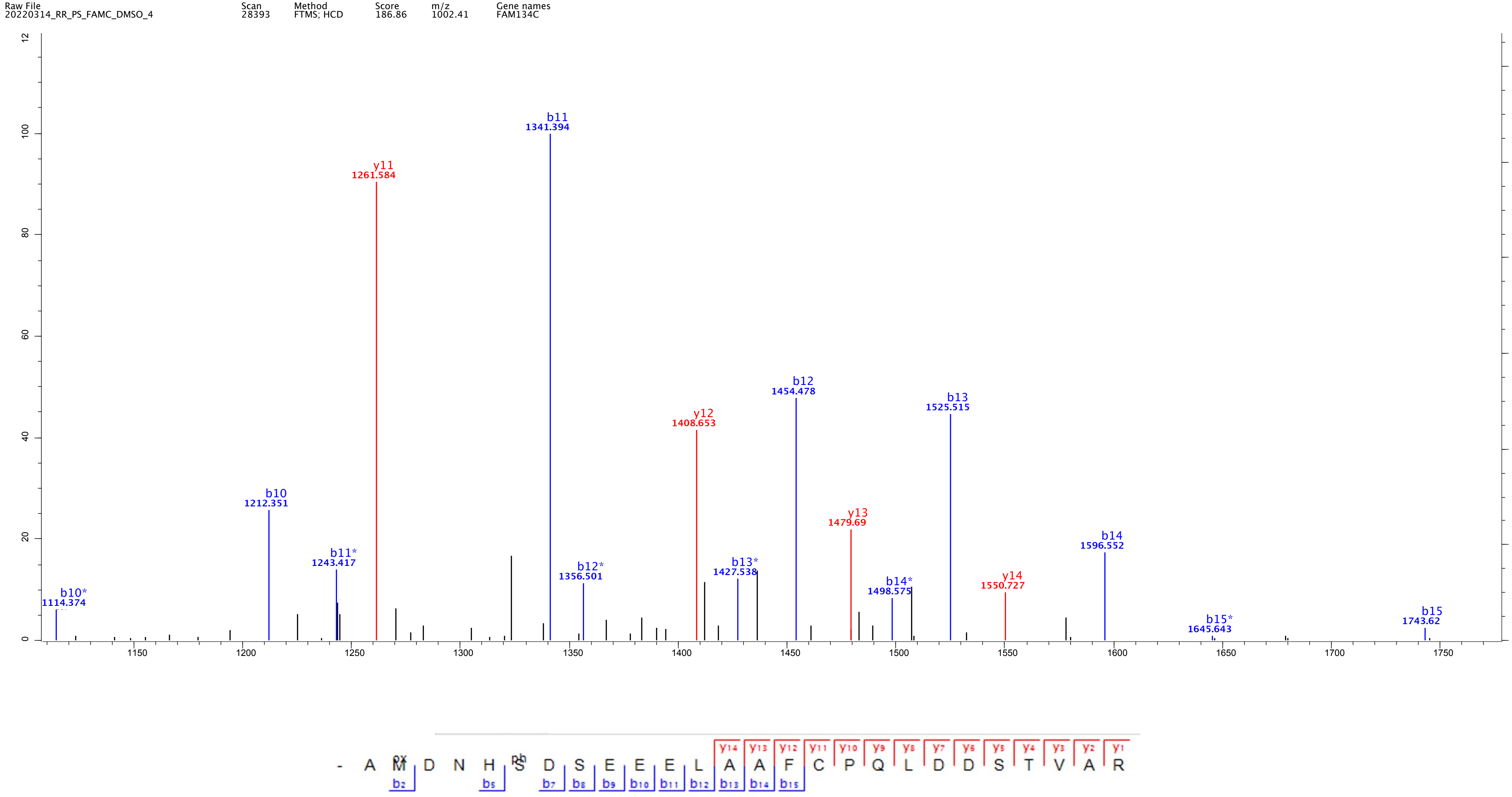

FAM134C-S260

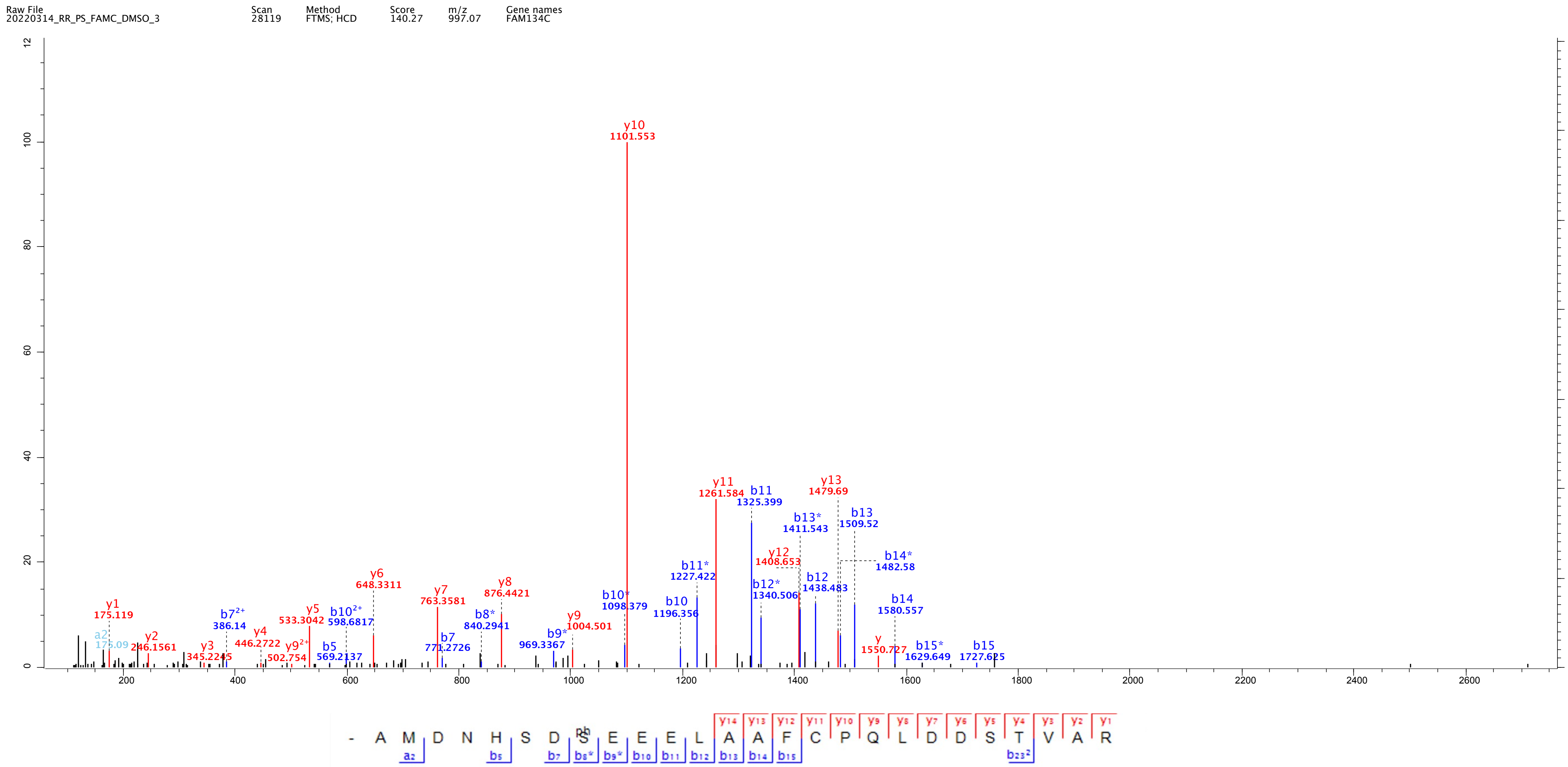

FAM134C-S260 – Low ions

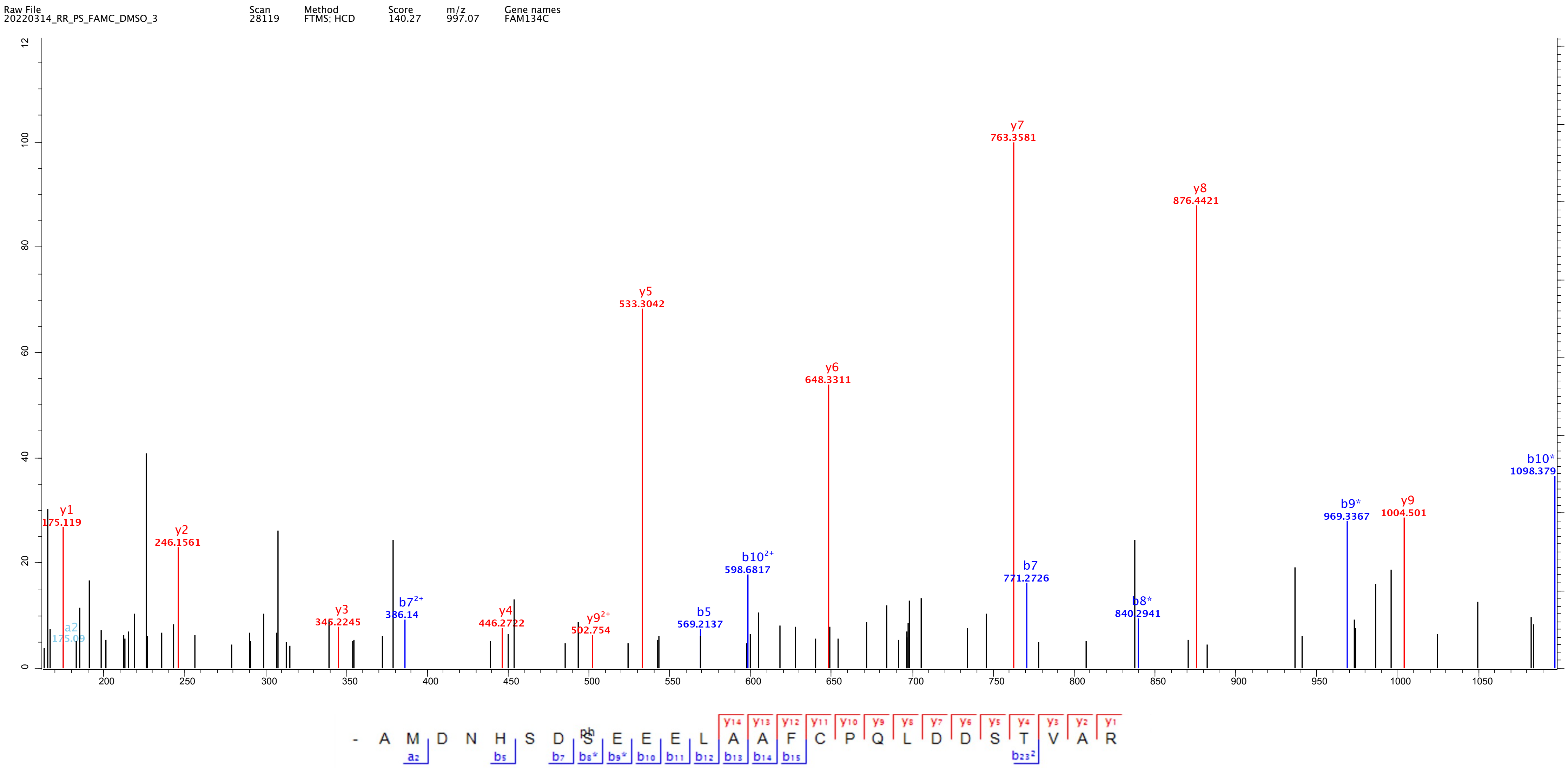

FAM134C-S260 – High ions

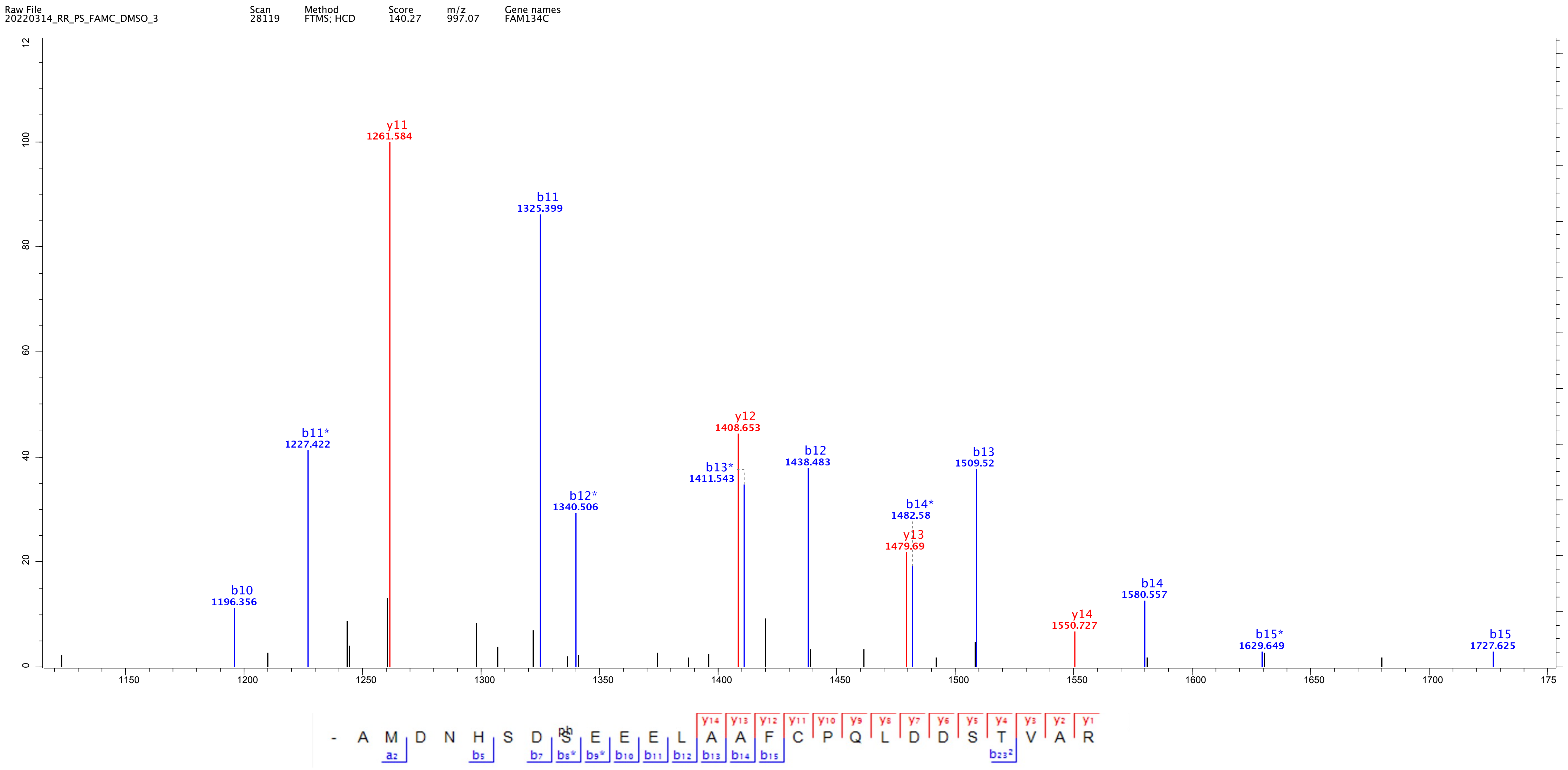

FAM134C-T283

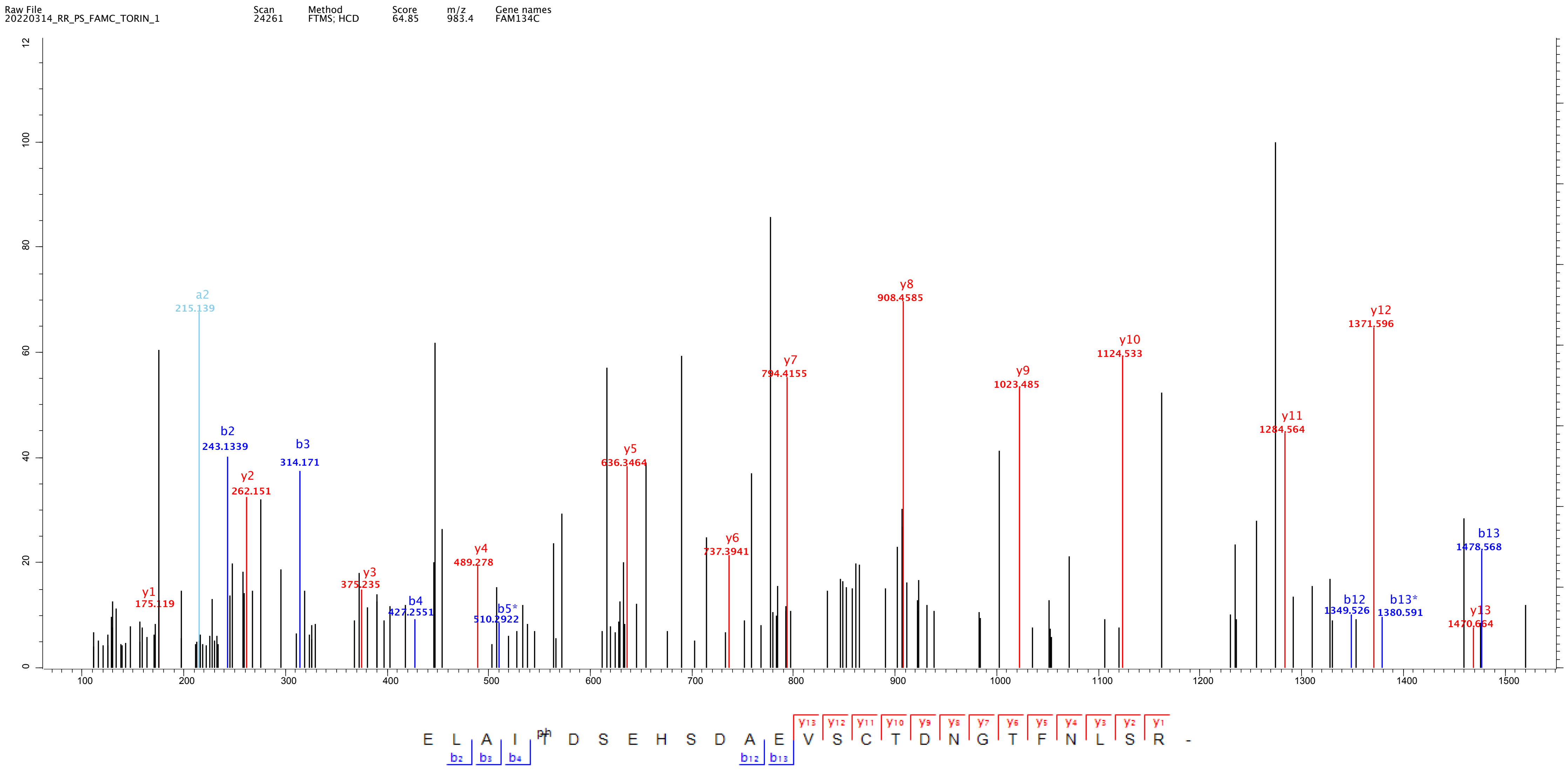

FAM134C-T283 – Low Ions

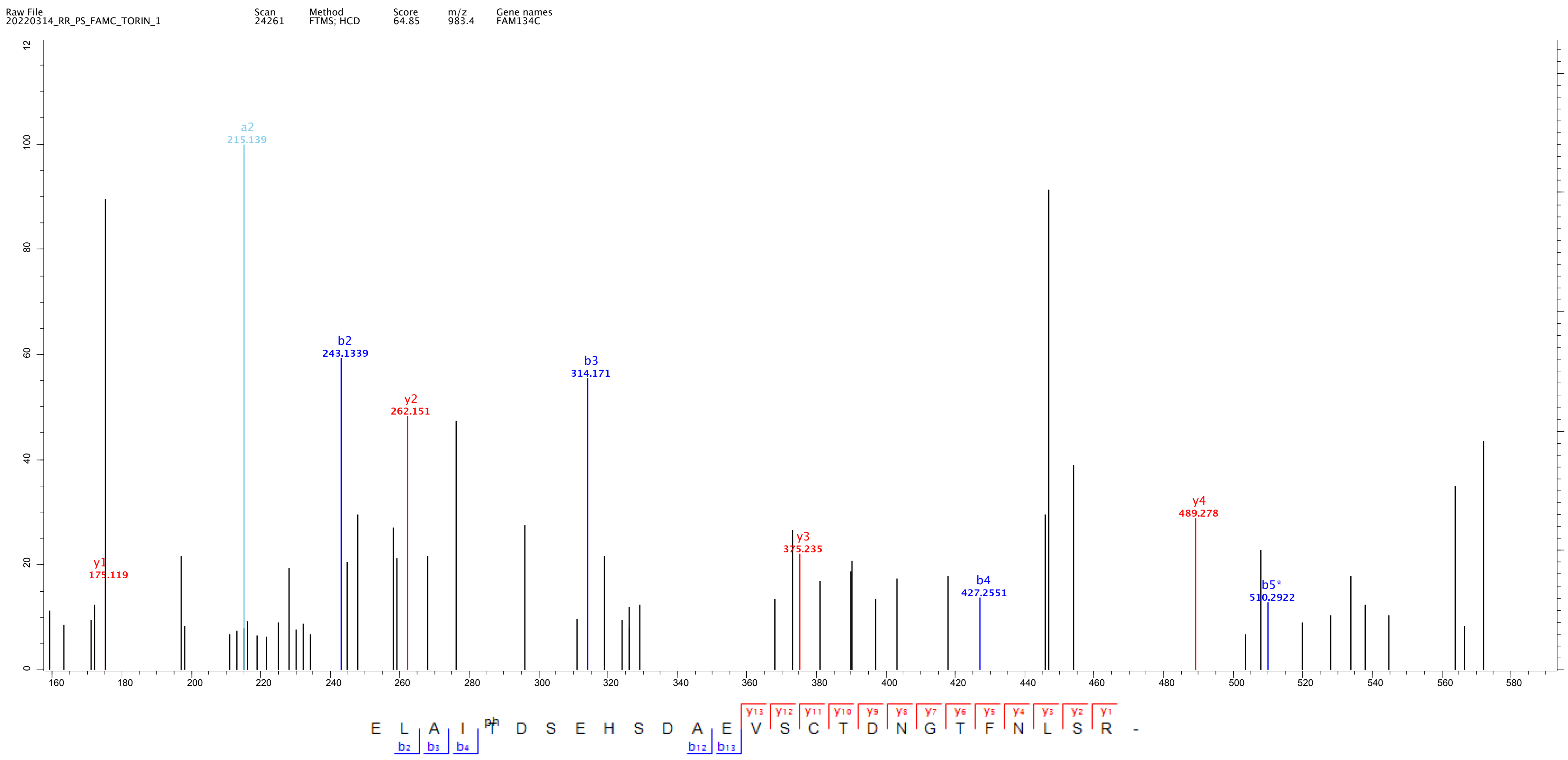

FAM134C-T283 – High Ions

Raw File	Scan	Method	Score	m/z	Gene names
20220314_RR_PS_FAMC_TORIN_1	24261	FTMS; HCD	64.85	983.4	FAM134C

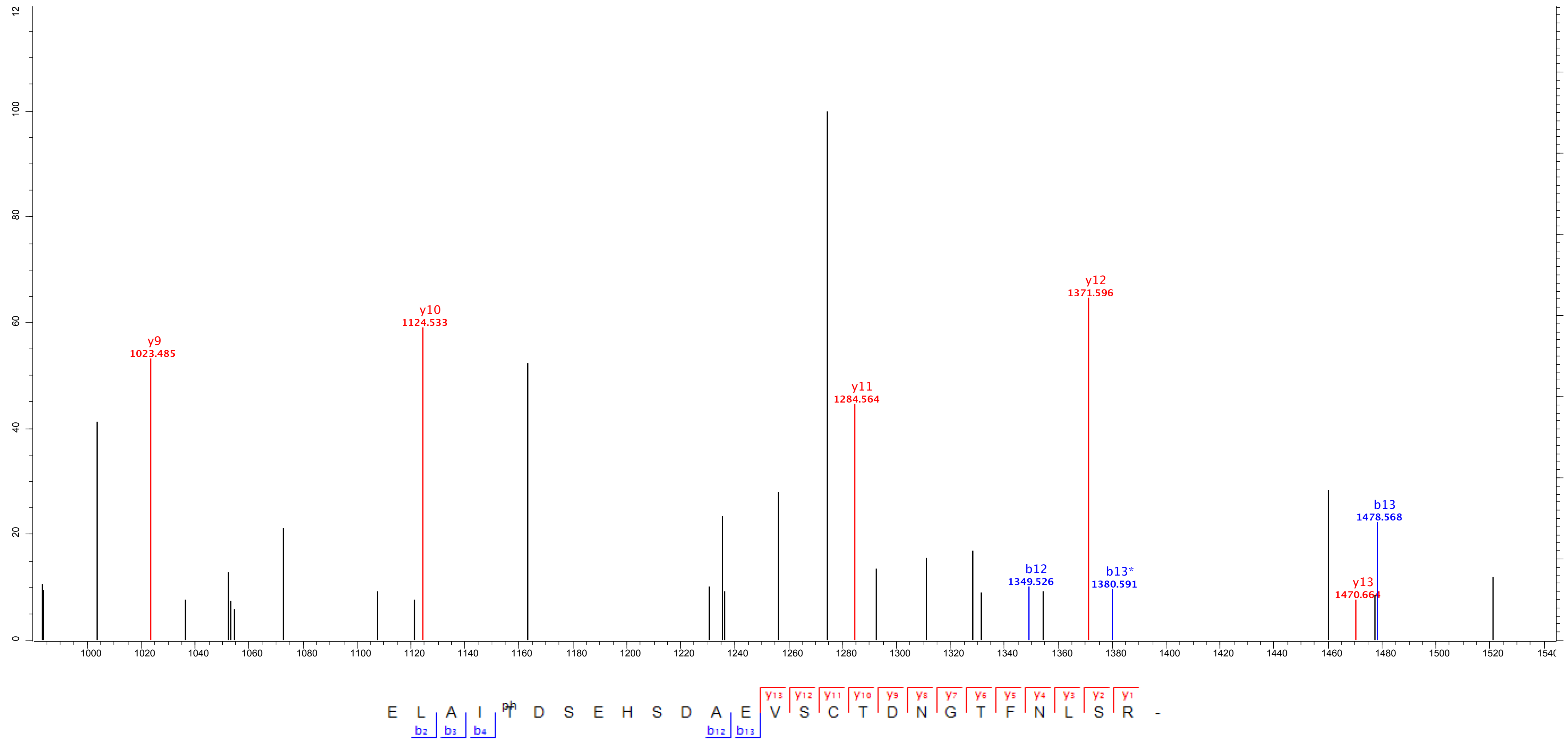

FAM134C-S285

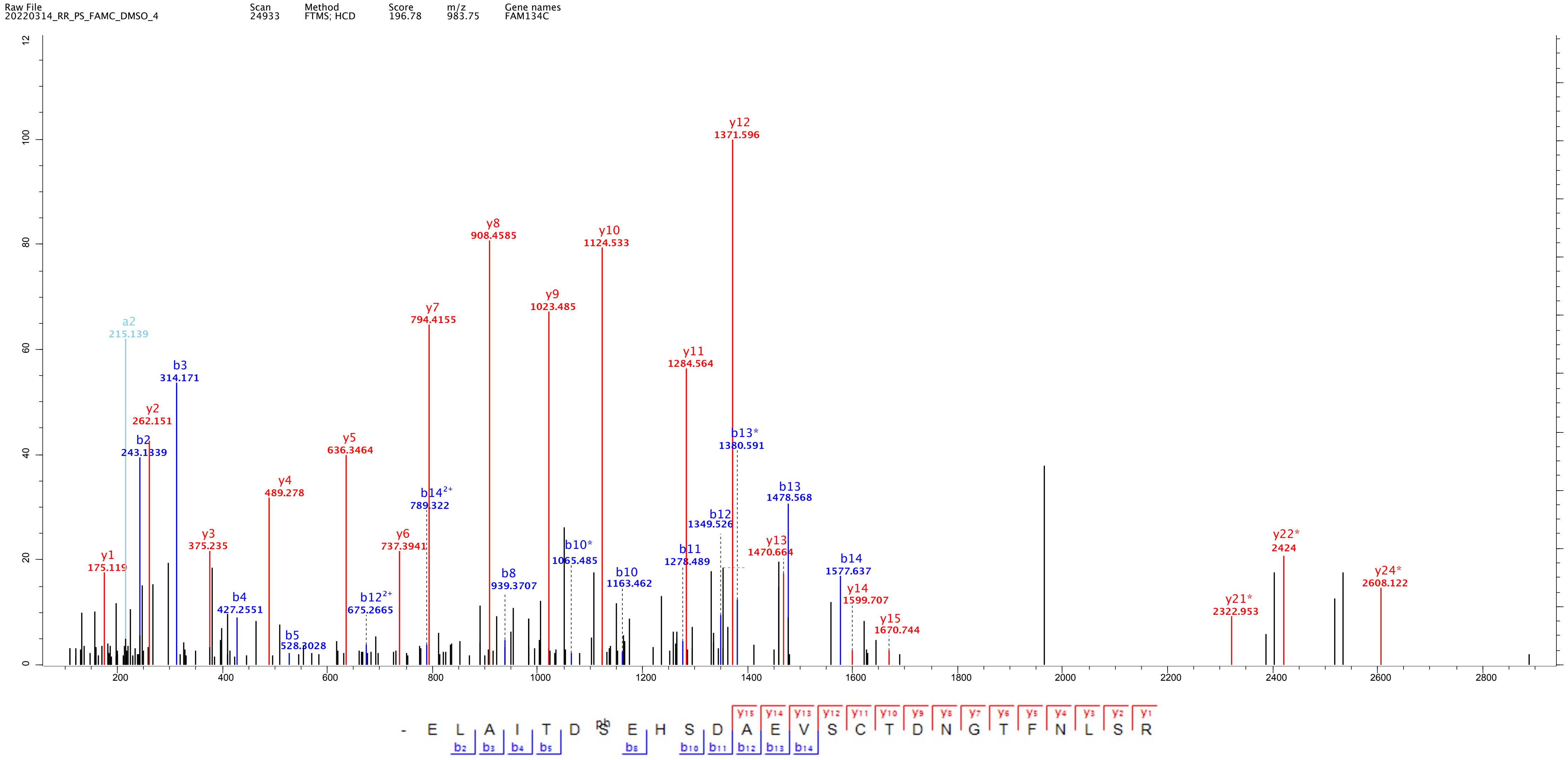

FAM134C-S285- Low Ions

Raw File	Scan	Method	Score	m/z	Gene names
20220314_RR_PS_FAMC_DMSO_4	24933	FTMS; HCD	196.78	983.75	FAM134C

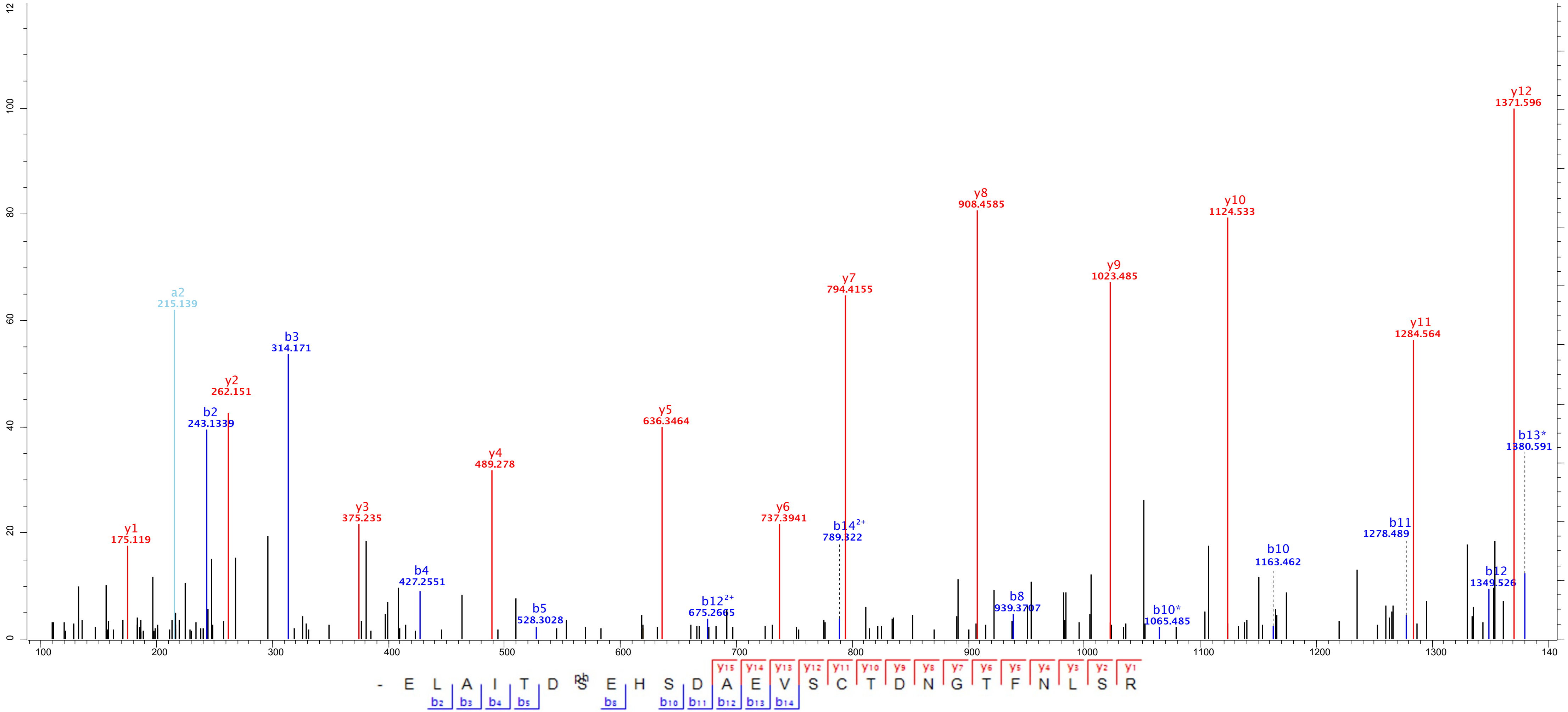

FAM134C-S288

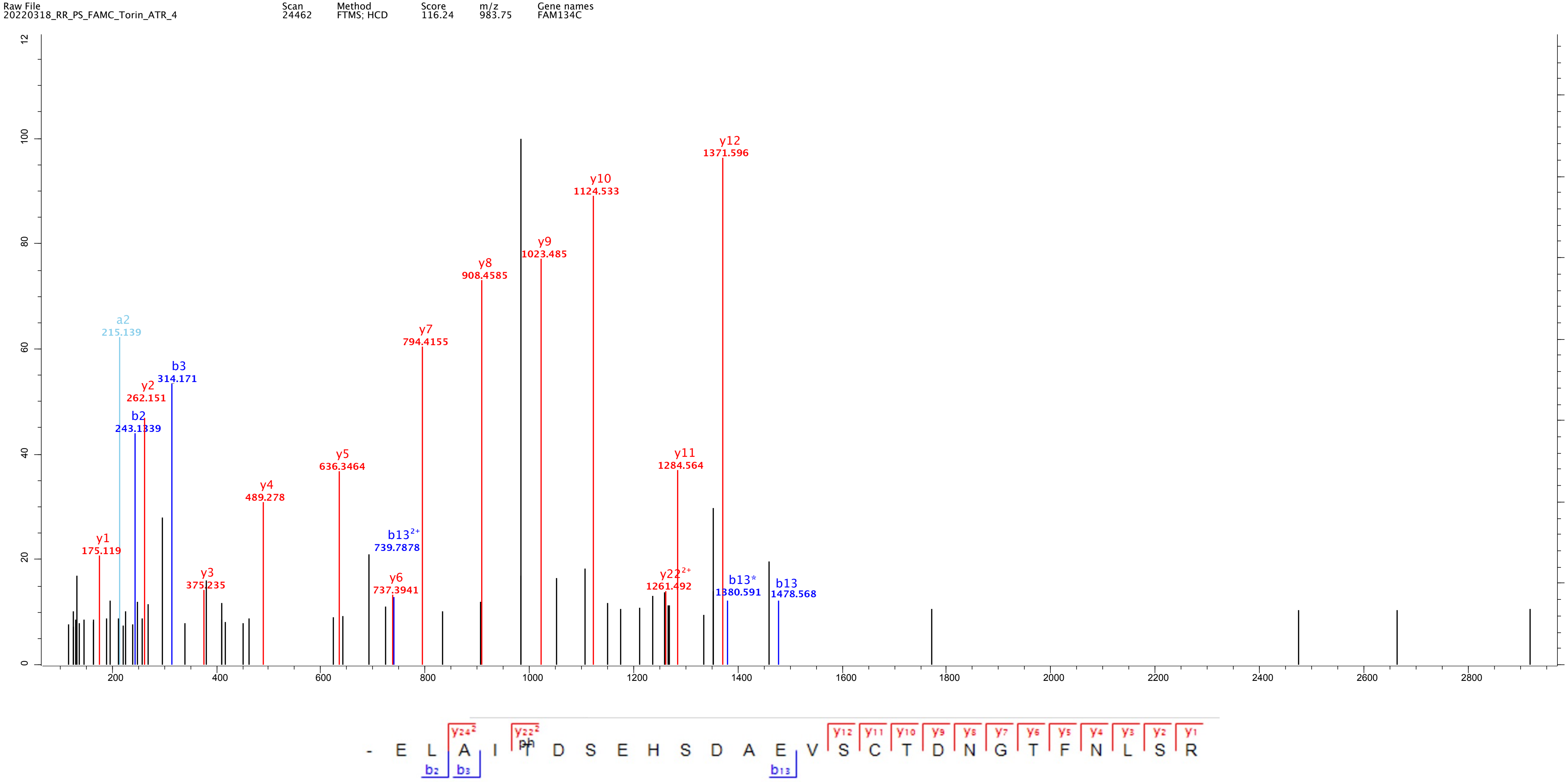

FAM134C-S313

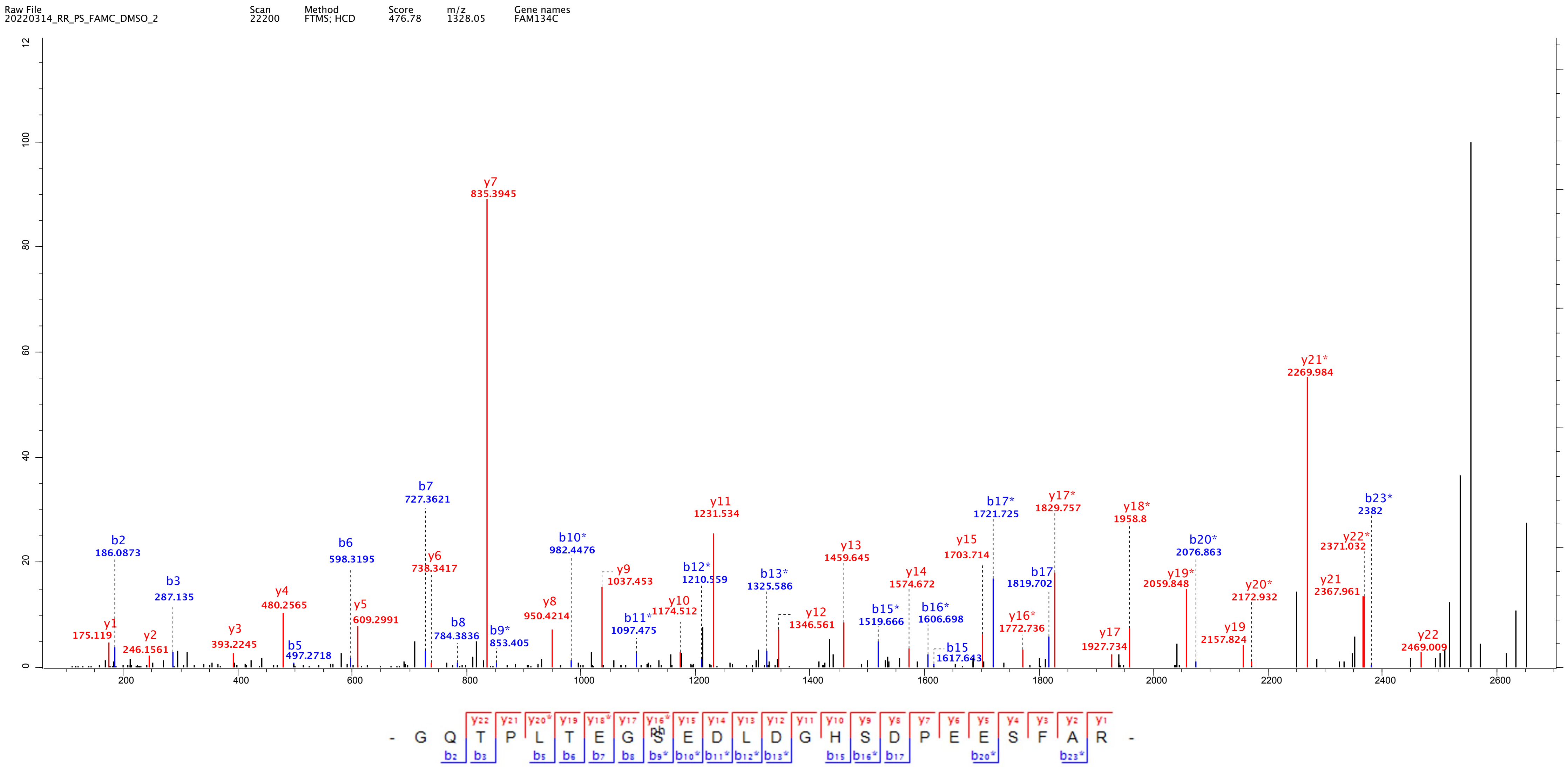

FAM134C-S313- Low ions

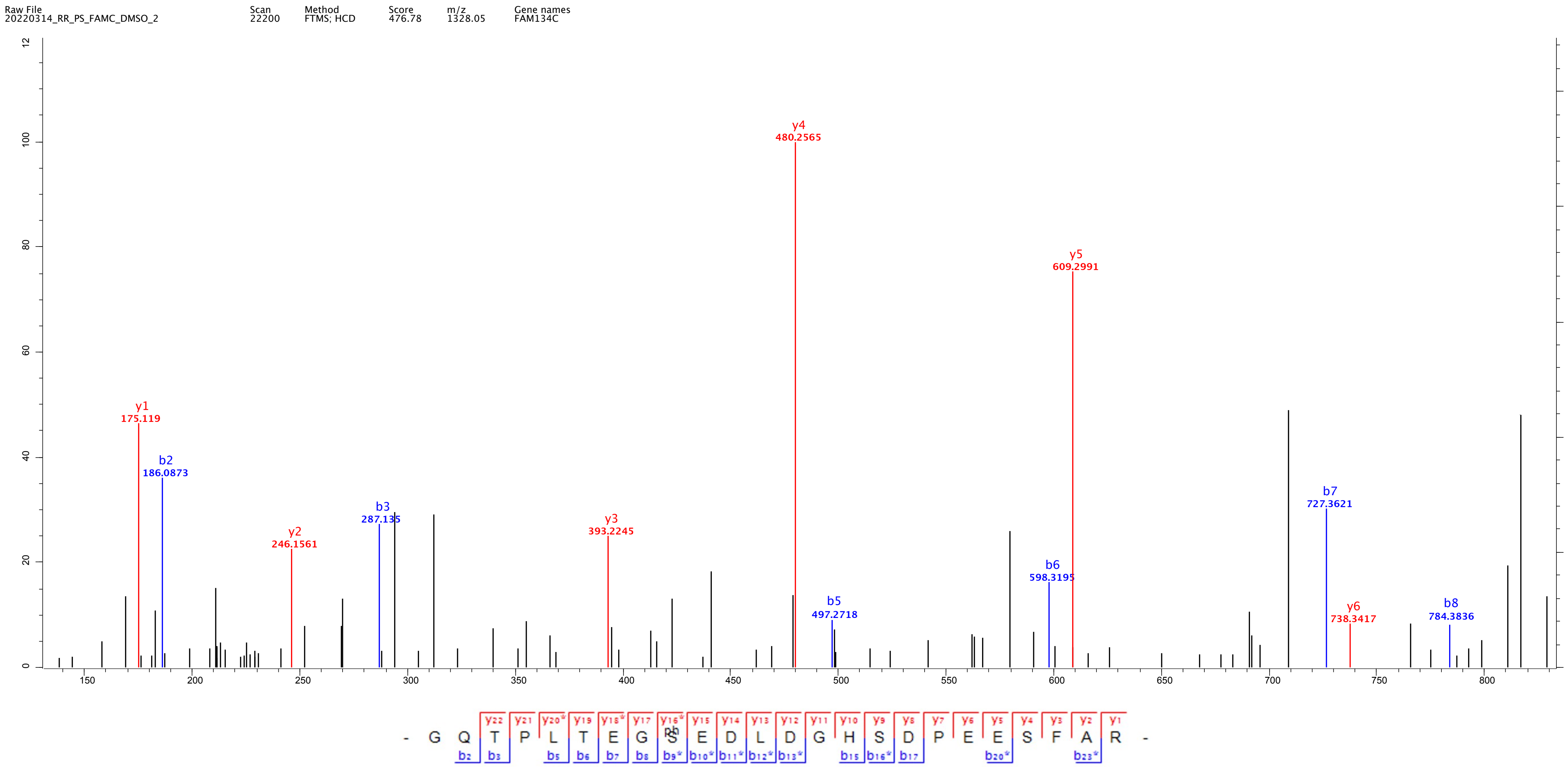

FAM134C-S313- Mid ions

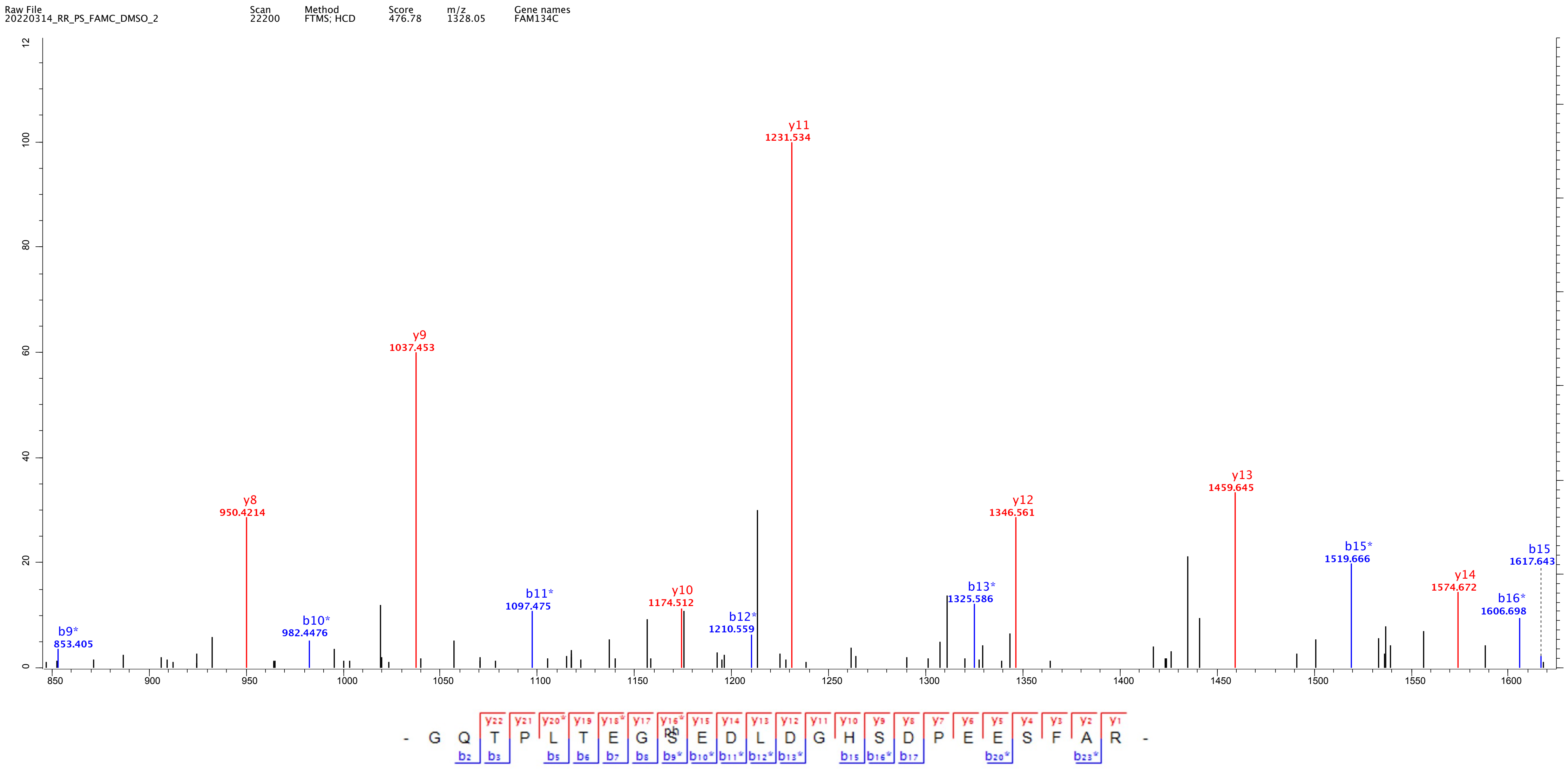

FAM134C-S313- High ions

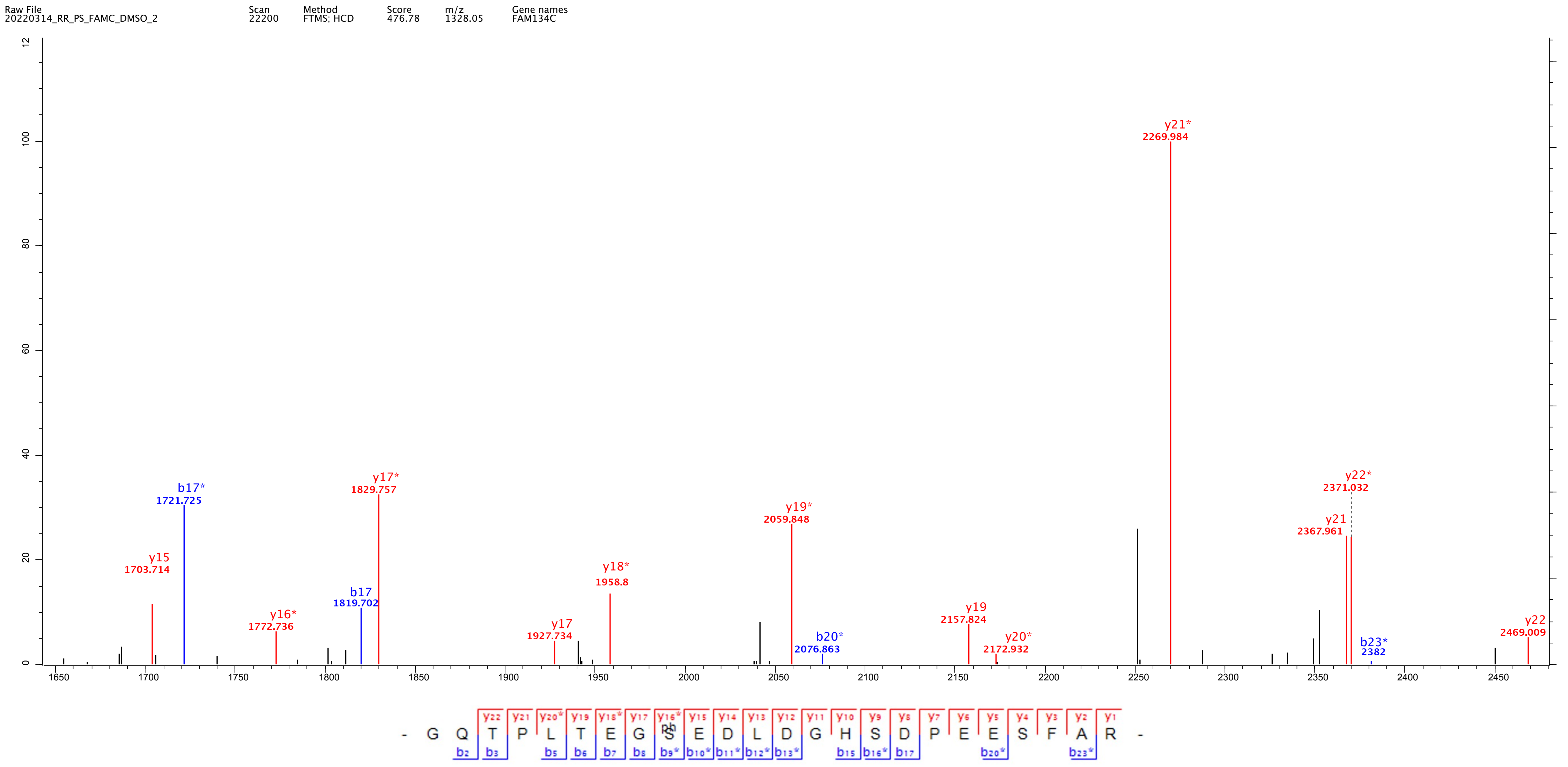

FAM134C-S320

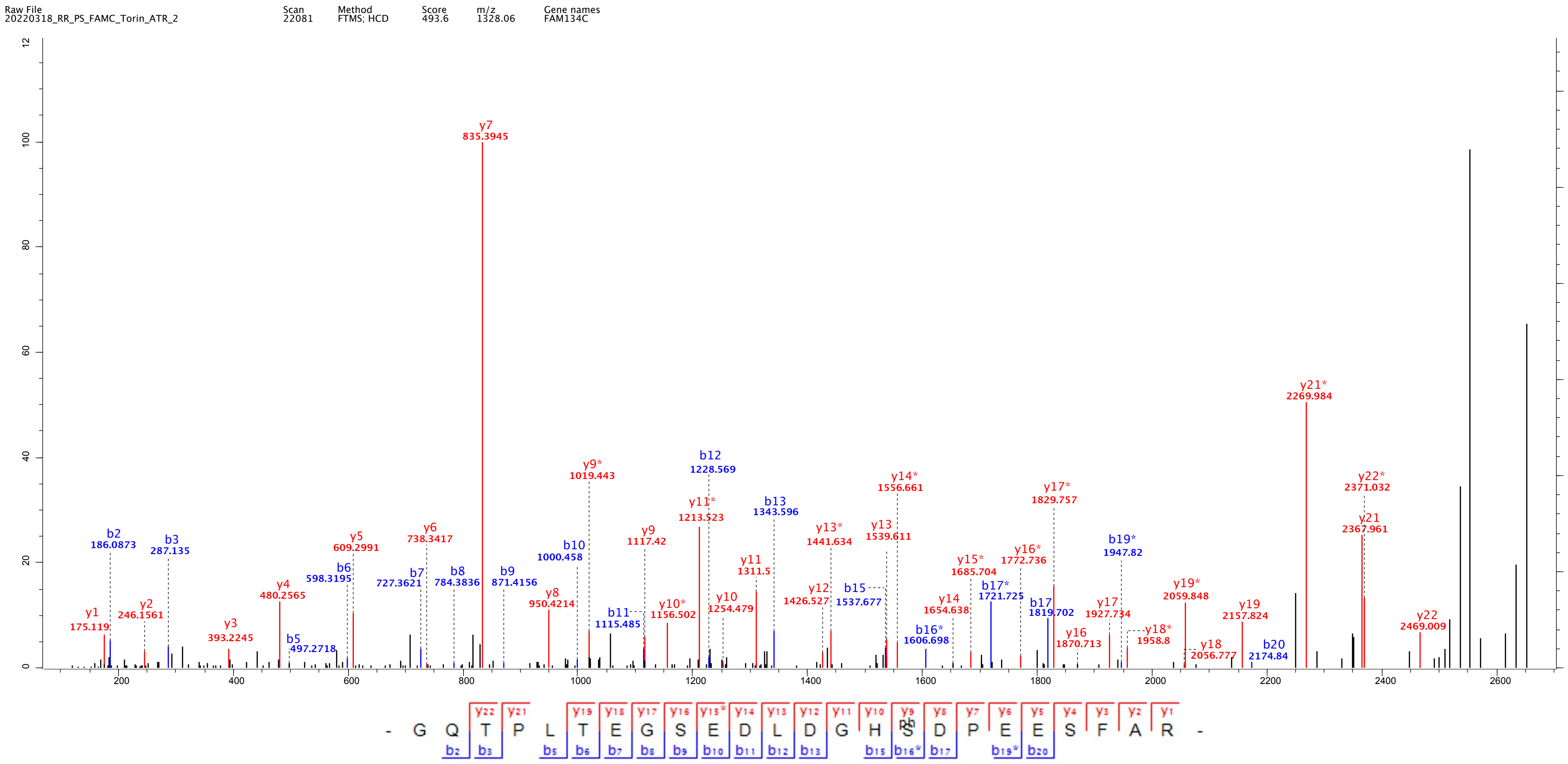

FAM134C-S320 – low Ions

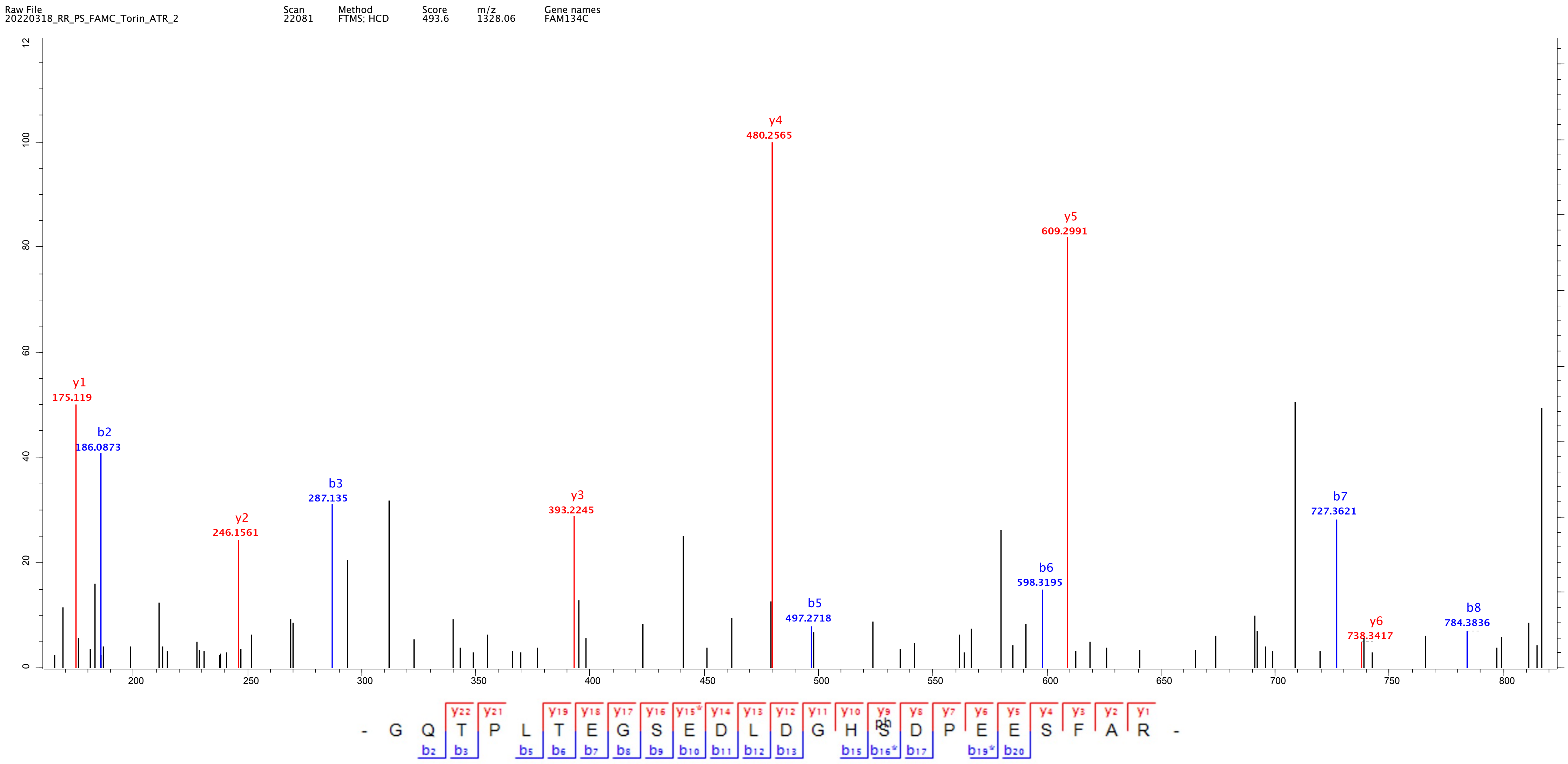

FAM134C-S320 – Mid Ions

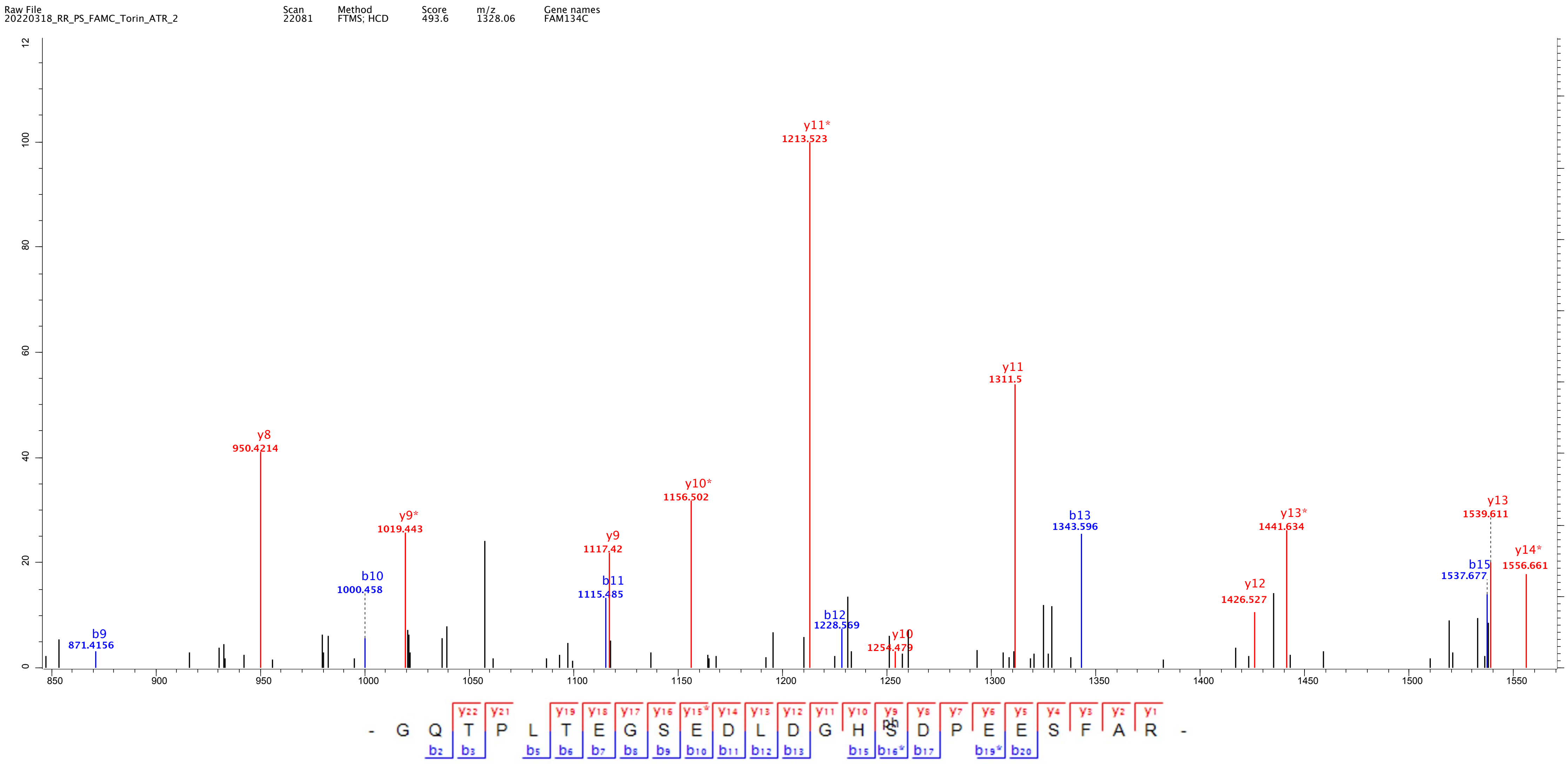

FAM134C-S320 – High Ions

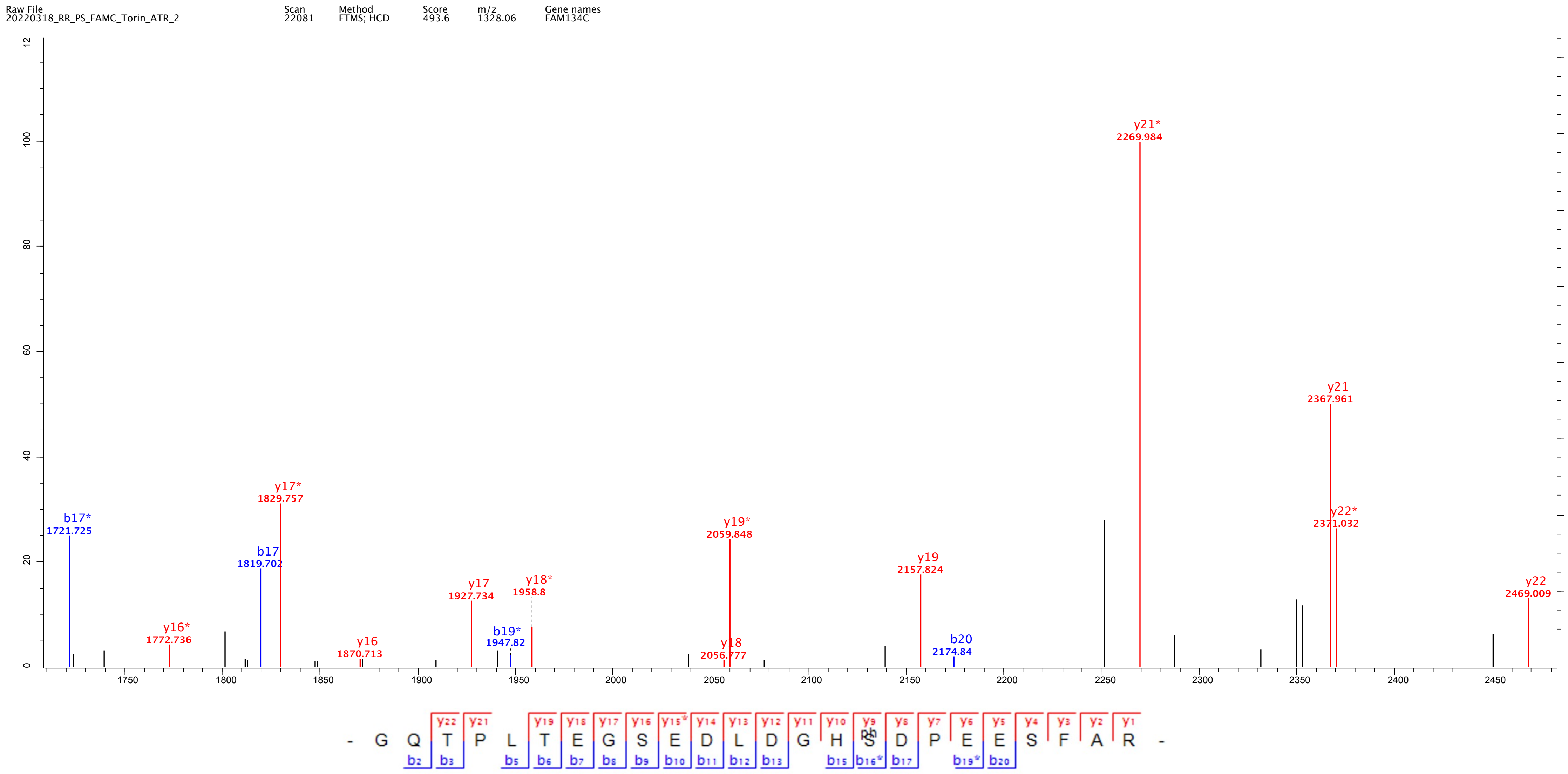

FAM134C-S360 – High Ions

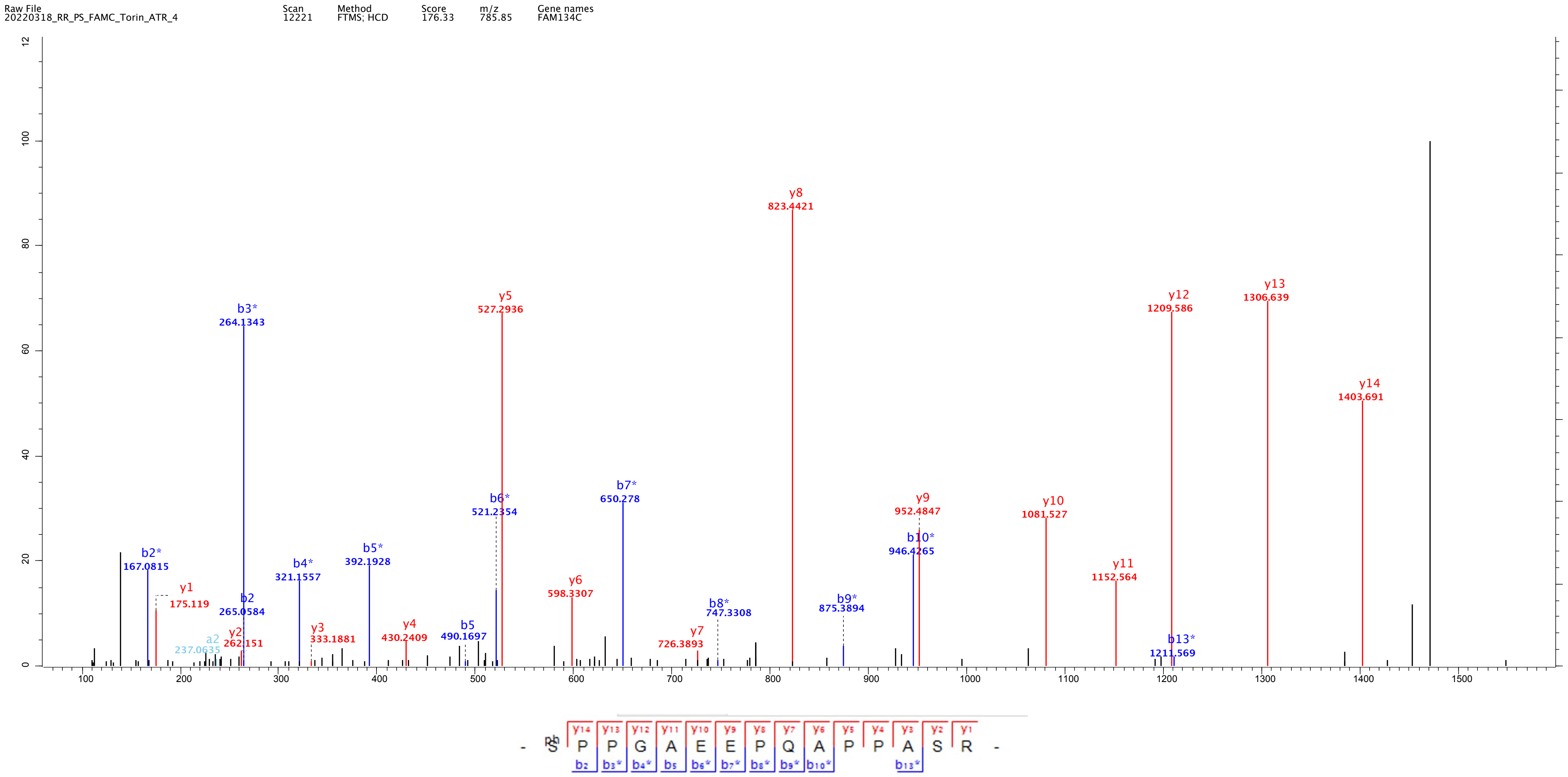

In Vitro FAM134B-S153

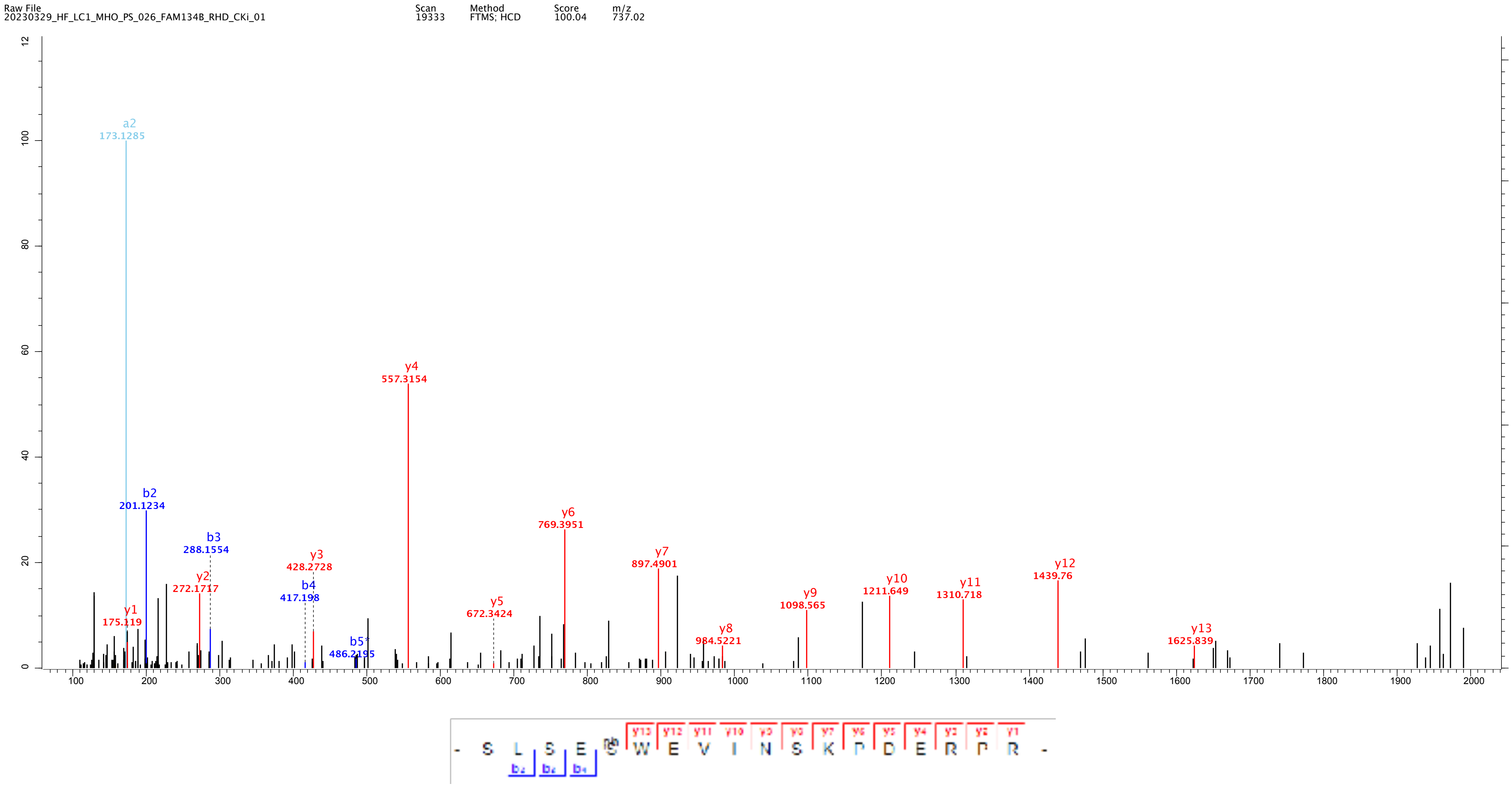

Supplement: Supplementary file 4 — Source Data [file 41467_2023_44101_MOESM4_ESM.zip › Source_Data_MS_Spectra.pdf]
